# Patient-reported financial toxicity among patients with liver cancer: a systematic review and meta-analysis

**DOI:** 10.3389/fpubh.2026.1958294

**Published:** 2026-10-15

**Authors:** Shangjin Li, Yuntong Liu, Yimeng Zhang, Lu Ren, Shujin Yue, Xiaoda Li, Fanping Meng, Chunxiang Su, Yiyuan Zhang, Jiaying Wang

**Affiliations:** 1 School of Nursing, Beijing University of Chinese Medicine, Beijing, China; 2 Medical and Health Analysis Center, Peking University, Beijing, China; 3 Senior Department of Infectious Diseases, The Fifth Medical Center of Chinese PLA General Hospital, Beijing, China

**Keywords:** liver cancer, financial toxicity, health-related quality of life, meta analysis, systematic review

## Abstract

**Objective:**

Financial toxicity (FT) refers to the negative impact of medical financial burden on patients’ overall health. The study aimed to quantify the prevalence and severity of patient-reported FT, identify the factors associated with FT in liver cancer patients, and thereby provide evidence to guide effective interventions to reduce the negative impacts of FT.

**Methods:**

In this study the following peer-reviewed databases were systematically searched: PubMed, Web of Science, Embase, the Cochrane Library, CINAHL, China National Knowledge Infrastructure, Wanfang Data, VIP, and Sinomed. The search was conducted from the inception of each database to March 2, 2026. Two independent authors extracted data using a standardized data extraction tool and assessed the methodological quality of included studies using the Joanna Briggs Institute Critical Appraisal Tools. A random-effects model was applied to generate pooled estimates of FT rates and severity.

**Results:**

Of the 13,412 articles, 39 were included in the study. Most studies were conducted in China (26/39, 66.67%). There was significant heterogeneity in the definition and measurement of FT. The pooled FT rate was 74% (95% CI: 61–85%), indicating high prevalence. The pooled FT score was 20.80 (95% CI: 19.70–21.92) for the COST (range 0–44) and 40.18 (95% CI: 27.12–53.24) for the European Organization for Research and Treatment of Cancer Quality of Life Questionnaire Core 30 Items (range 0–100), reflecting a moderate level of FT. These findings suggest that liver cancer patients present a high prevalence of FT, yet the overall severity remains moderately low.

**Conclusion:**

The findings of this study indicate that FT is a common but moderate issue in liver cancer care. However, the lack of standardized measurement tools and inconsistent reporting methods have impaired the comparability of available evidence. Therefore, future research should develop a validated FT assessment for liver cancer and test targeted interventions to improve care and policy.

**Systematic review registration:**

https://www.crd.york.ac.uk/PROSPERO/view/CRD420261345754, identifier: CRD420261345754.

## Introduction

Liver cancer is a formidable public health challenge worldwide, with extremely high incidence and mortality rates. It is the sixth most frequently diagnosed cancer and the third leading cause of cancer death (1). In 2020, an estimated 905,700 new cases of cancer and 830,200 related deaths were reported globally, accounting for 8.2% of all cancer-related mortality (2, 3). Clinical management remains challenging, as the majority of patients are diagnosed at the intermediate or advanced stage (4–6). Current therapeutic strategies for liver cancer include liver transplantation, resection, ablation, transarterial therapy, radiation therapy, and systemic therapy. The multidisciplinary treatment model results in complex and costly care, placing a substantial financial burden on patients and families (7, 8).

Financial toxicity (FT) refers to the negative impact of medical financial burden on patients’ psychological well-being, fiscal security, and overall health (9). FT consists of two key components, namely subjective financial distress and objective financial burden, which cover four distinct dimensions: direct medical costs, direct nonmedical costs, indirect costs, and psychological distress (10). Direct medical costs encompass hospitalization fees, pharmaceutical expenditures, laboratory charges, and outpatient treatment-related expenses. Direct nonmedical costs include transportation, accommodations, meals, and expenses arising from disease-related employment loss. Nonmedical and indirect expenses are generally not covered by insurance and financial aid programs, thus adding to patients’ out-of-pocket costs. Additionally, this psychological burden frequently manifests as financial worry among patients and families (9–11).

FT is notably prevalent and severe among patients with liver cancer (12, 13). Prolonged multimodal treatment for liver cancer imposes substantial cumulative out-of-pocket costs (14). An economic study conducted in China revealed that hepatocellular carcinoma patients incurred an average annual cost of 33,044 RMB (15). However, FT is inherently subjective (9). Objective medical costs alone cannot fully capture the experience of financial stress, nor do they adequately reflect its impact on quality of life. Patient-reported data therefore provide an important approach for the quantitative assessment of FT (16, 17).

Patient-reported outcome measures (PROMs) enable patients to self-report their experiences of financial distress and the impact of financial burden on quality of life (18). Currently, a variety of PROMs have been developed and validated, both domestically and internationally, to assess FT in cancer patients. The Comprehensive Score for Financial Toxicity-Functional Assessment of Chronic Illness Therapy (COST) was developed by DeSouza et al. in 2014 as a standardized PROM for assessing FT in cancer patients, and has been validated in diverse cancer populations (19). In addition, other financial stress measures have been widely used, including the Breast Cancer Finances Survey Inventory (20), the Socioeconomic Well-being Scale (21), and the InCharge Financial Distress/Financial Well-being Scale (22). Beyond FT-specific instruments, financial burden has also been assessed using financial difficulty items embedded in broader quality-of-life or social-difficulty measures, such as the Social Difficulties Inventory (23), the Cancer Care Outcomes Research (24), and the European Organization for Research and Treatment of Cancer Quality of Life Questionnaire Core 30 Items (EORTC QLQ-C30) (25).

In patients with liver cancer, some studies have also employed the PROMs to evaluate FT. Zhou and Liu (26) found that the average COST scores of patients with liver cancer were (22.94 ± 6.33), reflecting a moderate level of FT. Sun et al. (27) showed that liver cancer patients had a mean economic difficulty score of (30.98 ± 30.56) on the EORTC QLQ-C30. Besides quantitative measures, some studies have employed qualitative approaches to explore patients’ experiences of financial hardship, providing in-depth insights into the nature and impact of FT that standardized instruments may not fully capture. Wu et al. (28) reported that, based on qualitative interviews, the FT experience of patients with primary liver cancer was profoundly influenced by multi-level factors. Overall, patient-reported research on FT in liver cancer has expanded considerably.

However, there is currently a lack of systematic summary of patient-reported studies on FT in liver cancer patients, as the available evidence is highly scattered across various single-center, small-sample studies. The prevalence and burden of FT remain unclear due to a lack of comprehensive pooled analysis. Therefore, this systematic review and meta-analysis aims to quantify the prevalence and severity of patient-reported FT, identify the factors associated with FT in liver cancer patients, and thereby provide evidence to guide effective interventions to reduce the negative impacts of FT.

## Method

The systematic review protocol followed the Preferred Reporting Items for Systematic Reviews and Meta-Analyses guidelines (PRISMA) and was registered in the PROSPERO database under the registration number CRD420261345754.

### Search strategy

The literature search was performed in two phases: an initial search from database inception to September 12–15, 2025, and an update was performed on March 2, 2026. Two authors (LSJ and LYT) systematically searched the following peer-reviewed databases: PubMed, Web of Science, Embase, the Cochrane Library, CINAHL, China National Knowledge Infrastructure (CNKI), Wanfang Data, VIP, and Sinomed. A broad search strategy was designed specifically for the peer-reviewed literature, using search terms related to liver cancer (e.g., “liver cancer,” “liver neoplasms”) and FT (e.g., “financial toxicity,” “financial stress,” “quality of life”). The detailed search strategies for the above databases are provided in Supplementary file 1. In addition, the snowballing technique was applied by examining the reference lists of initially included studies to identify further relevant publications.

### Eligibility criteria

The eligibility criteria were as follows: (1) FT related to liver cancer diagnosis and treatment; (2) an analysis of FT rates, severity, and FT experience for the diagnosis and treatment of liver cancer; (3) studies providing data on FT definitions, measurements, or associated factors; (4) quantitative and qualitative studies; (5) language was limited to Chinese and English.

The exclusion criteria included: non-original research; non-human subjects; clinical trials; case report or trial methodologies; duplicate publications; or analyses of objective out-of-pocket costs using institutional or insurance databases, since such analyses are not based on patient-reported data.

### Study selection and data extraction process

Records identified from database searches were de-duplicated and then exported for screening in EndNote 20. Titles and abstracts were independently assessed by two reviewers (LSJ and ZYY). Full texts were retrieved if they met the eligibility criteria. Discrepancies were resolved by consensus.

Data extraction was performed independently by two authors (LSJ and WJY) using a piloted, standard data extraction form. Any differences in opinion were resolved through discussion or consultation with a third party (Su CX). Data extracted included authors, publication year, location, study design, outcome measurement criteria, sample size, measurement of FT, cancer type, stage system, treatment setting, phenomenon of interest, methodological quality assessment, main outcomes, and factors associated with FT.

FT outcome measures were grouped into four categories: (1) validated FT-specific instruments; (2) financial-difficulty items from broader quality-of-life instruments; (3) objective measures of financial burden derived from patient surveys or interviews; and (4) qualitative assessments of financial hardship. Summary estimates were sought, and data were extracted for the entire cohort and specific subgroups. For studies presenting disaggregated data, these figures were extracted separately when possible.

### Risk of bias assessment

Two researchers (LSJ and ZYM) independently assessed the methodological quality of the included studies. Qualitative studies were assessed using the Joanna Briggs Institute Critical Appraisal Checklist for Qualitative Research. Quantitative studies were assessed using the Joanna Briggs Institute Critical Appraisal Checklist, corresponding to each study design, including cross-sectional and cohort studies. Disagreements were resolved through discussion or consultation with a third party (Su CX). Scores of 1 were assigned for “yes,” 0 for “no,” and “U” for “unclear.” These scores were converted into percentages to determine the risk of bias, with ≤ 49% indicating high-risk, 50–69% indicating moderate risk, and ≥ 70% indicating low risk (29).

### Definitions

FT rates were derived based on the definition and reporting criteria specified in each study. In studies that measured patient-reported severity only, ratings were reported on a 2-point (1 = yes, 2 = no) or 4-point (1 = none, 2 = somewhat, 3 = quite a bit, 4 = a lot) scale. To calculate the FT rate from these studies, FT is defined as a moderate-to-severe rating (score of 1 on a 2-point scale or 3–4 on a 4-point scale), because these cutoffs were applied consistently to determine FT among the included studies (30–32). The proportion of participants above the cutoff determined the FT rate in these studies.

### Data analysis

RevMan 5.4 and Stata 18 software were used for data synthesis. For continuous variables, the mean difference (MD) or standardized mean difference (SMD) was calculated; for dichotomous variables, the odds ratio (OR) or risk difference (RD) was used as the effect measure; for single-arm prevalence meta-analysis, the pooled proportion was used. The pooled effect estimates were reported with their 95% confidence intervals (95% CI), and a two-sided *p*-value <0.05 was considered statistically significant. Heterogeneity was assessed by Cochran’s *Q* test (*χ*^2^) and the *I*^2^ statistic. The *I*^2^ statistic was interpreted as follows: <25% indicated low heterogeneity, 25–75% indicated moderate heterogeneity, ≥75% indicated substantial heterogeneity. A fixed-effect model was applied if *I*^2^ < 75%; otherwise, a random-effect model was used. All analyses in this study used a random-effect model because of the considerable heterogeneity among studies.

To explore potential sources of heterogeneity, sensitivity analysis and subgroup analysis were conducted based on relevant factors. Countries were categorized by income level using the World Bank classification for fiscal year 2026 (33). Publication bias was initially assessed using a funnel plot and then evaluated statistically using Begg’s test and Egger’s test. Furthermore, the trim-and-fill method was used to adjust for any potential publication bias. Qualitative analysis was used as contextual evidence and described the phenomena of interest.

## Results

A total of 13,412 articles were identified from the initial search. After removing 2,801 duplicates and 10,027 irrelevant studies by screening titles and abstracts, 584 full-text articles were further checked. Ultimately, 39 studies were included in this review, of which 2 were identified by manually searching the reference lists of identified studies (Figure 1).

**Figure 1:**
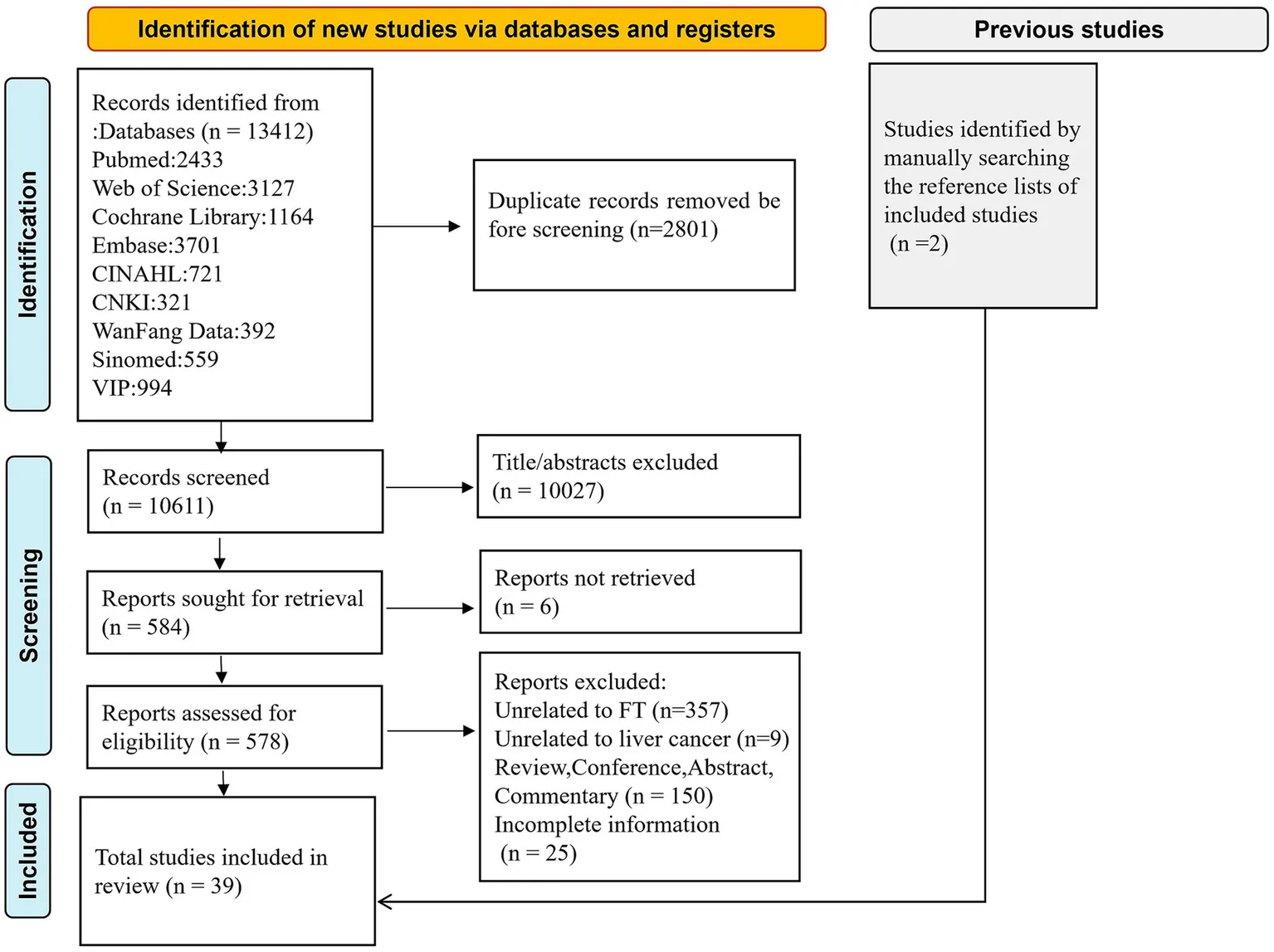
PRISMA flow diagram.

The included studies were published between 2002 and 2026, with a steady increase in the number of publications from 2023 to 2025. The majority were conducted in China (26/39, 66.67%), followed by the United States (4/39, 10.26%). The publications comprised 26 English and 13 Chinese articles. Among the included studies, 18 (46.15%) were cross-sectional studies, 11 (28.21%) were longitudinal studies, and 10 (25.64%) were qualitative studies. The sample sizes varied considerably, ranging from 12 to 2,223 participants. The included studies varied considerably in terms of treatments and the cancer stages of patients. The methodological quality of the included studies was generally high. All cross-sectional and qualitative studies scored above 70%, indicating good quality. Cross-sectional studies scored lowest on the item concerning strategies for confounding factors. Qualitative studies scored lowest on the item addressing researcher influence. Among the 11 longitudinal studies, only 3 (27.27%) achieved scores above 70%. Relatively low scores were observed on five items: group similarity and recruitment, exposure measurement, confounding strategies, baseline outcome status, and strategies for incomplete follow-up, indicating poor methodological quality. Detailed characteristics are presented in Table 1, Supplementary file 3.

**Table 1:** Basic characteristics of included studies.

Author (year)	Country/region	Study design	Outcome measures/phenomena of interest	Sample size	Cancer type	Stage system	Treatment setting	Quality assessment (score)
Zou et al. (37) (2025)	China	Cross-sectional study	PROM (COST)	495	PLC	TNM class	Systemic therapy; surgical therapy; others	8
Tsai et al. (57) (2026)	China	Cross-sectional study	PROM (EORTC QLQ-C30); HADS	101	HCC	TNM class	Surgical therapy; locoregional therapy	8
Li and Zou (32) (2025)	China	Cross-sectional study	PROM (COST); PROM (EORTC QLQ-C30)	200	PLC	Not reported	Surgical therapy; others	8
Zou (12) (2025)	China	Cross-sectional study	PROM (COST); FT rate	240	PLC	Not reported	Locoregional therapy	8
Mao et al. (13) (2026)	China	Cross-sectional study	PROM (COST); FT rate	268	PLC	CNLC class	Not reported	8
Lee et al. (14) (2025)	United States	Cross-sectional study	PROM (COST); FT rate	38	HCC	BCLC class	Locoregional therapy	8
Zhang et al. (36) (2025)	China	Cross-sectional study	PROM (COST-FACIT-V2); SPBS-CP	236	PLC	TNM class	Multiple treatments	7
Liu et al. (35) (2025)	China	Cross-sectional study	PROM (COST)	239	HCC	TNM class	Surgical therapy; locoregional therapy; systemic therapy	8
Sun et al. (27) (2025)	China	Cross-sectional study	PROM (EORTC QLQ-C30)	752	PLC	CNLC class	Systemic therapy	8
Zhou and Liu (26) (2024)	China	Cross-sectional study	PROM (COST)	265	HCC; ICC; HCC-CCA	Not reported	Surgical therapy; locoregional therapy; systemic therapy	8
Wu et al. (34) (2024)	China	Cross-sectional study	PROM (COST); FT rate	251	PLC	TNM class	Locoregional therapy	8
Zhu et al. (30) (2024)	China	Cross-sectional study	PROM (COST); FT rate; CD-RISC	362	PLC	Not reported	Locoregional therapy; systemic therapy; surgical therapy	7
Lei et al. (38) (2020)	China	Cross-sectional study	FT rate	2,223	HCC; Others	TNM class	Systemic therapy; locoregional therapy	6
Li et al. (62) (2019)	Hong Kong SAR, China	Cross-sectional study	PROM (EORTC QLQ-C30)	472	HCC	Child-Pugh class	Surgical therapy; locoregional therapy; Systemic therapy	8
Li et al. (63) (2019)	Hong Kong SAR, China	Cross-sectional study	PROM (EORTC QLQ-C30)	445	HCC	BCLC class	Surgical therapy; locoregional therapy; systemic therapy	8
Chie et al. (64) (2017)	United Kingdom; Italy; Japan; France; Taiwan, China; Hong Kong SAR, China	Cross-sectional study	PROM (EORTC QLQ-C30)	227	HCC	BCLC class	Surgical therapy; locoregional therapy; systemic therapy; off-treatment	8
Tang et al. (65) (2016)	China	Cross-sectional study	PROM (EORTC QLQ-C30)	218	HCC	Not reported	Surgical therapy; locoregional therapy; systemic therapy; other treatments	6
Che et al. (15) (2016)	China	Cross-sectional study	A questionnaire collects information on medical costs and non-medical costs	131	HCC	Not reported	Not reported	6
Li et al. (66) (2025)	Hong Kong SAR, China	Longitudinal study	PROM (EORTC QLQ-C30)	318	HCC	TNM class, BCLC class	No active antitumor treatment	9
George et al. (67) (2025)	India	Longitudinal study	PROM (EORTC QLQ-C30)	40	HCC	BCLC class	Locoregional therapy	7
Ong et al. (68) (2025)	Philippines	Longitudinal study	PROM (EORTC QLQ-C30)	19	HCC	BCLC class	Locoregional therapy	6
Shomura et al. (69) (2024)	Japan	Longitudinal study	PROM (EORTC QLQ-C30)	58	HCC	BCLC class	Surgical therapy; locoregional therapy; systemic treatment; off-treatment	8
van Roekelet al (70). (2020)	Netherlands	Longitudinal study	PROM (EORTC QLQ-C30)	53	Liver metastases	Not reported	Not reported	9
Kim et al. (71) (2020)	South Korea	Longitudinal study	PROM (EORTC QLQ-C30)	31	HCC	mUICC class	Surgical therapy; locoregional therapy; combination treatment	7
Meier et al. (72) (2015)	United States	Longitudinal study	PROM (EORTC QLQ-C30)	130	HCC	BCLC class	No active antitumor treatment	8
Zhang (73) (2012)	China	Prospective longitudinal	PROM(EORTC QLQ-C30)	54	HCC	Edmondson Steiner	Surgical therapy	6
Romero et al. (74) (2008)	Netherlands	Longitudinal study	PROM (EORTC QLQ-C30)	25	Liver metastases; HCC	Child-Pugh	Locoregional therapy	6
Dasgupta et al. (75) (2008)	United Kingdom	Longitudinal study	PROM (EORTC QLQ-C30)	130	Liver metastases; HCC; Others	Not reported	Surgical therapy	7
Brans et al. (25) (2002)	Belgium, Italy	Longitudinal study	PROM (EORTC QLQ-C30)	20	HCC	TNM class	Locoregional therapy	7
Han et al. (76) (2026)	China	Qualitative study	The financial burden experiences of liver cancer patients receiving targeted therapy in combination with immunotherapy.	15	Liver cancer	TNM class	Systemic therapy	10
Wu et al. (28) (2025)	China	Qualitative study	The experience of FT among patients with primary liver cancer.	19	PLC	Not reported	Not reported	9
Lu et al. (46) (2025)	China	Qualitative study	The attitude of patients with liver cancer toward clinical drug trials.	19	PLC	Not reported	Antineoplastic drugs	10
Sun et al. (77) (2024)	China	Qualitative study	The continuum of care needs for patients undergoing immunotherapy for liver cancer.	17	Liver cancer	Not reported	Systemic therapy	9
Wörns et al. (47) (2024)	United States; France; Germany; Japan	Qualitative study	The patient experience of liver cancer in relation to treatment options, treatment decision-making, and treatment goals throughout the disease journey.	50	HCC	BCLC class	Surgical therapy	10
Yu et al. (78) (2023)	China	Qualitative study	Understanding the symptom burden and unmet supportive care needs in advanced liver cancer patients following interventional procedures.	25	Liver cancer	TNM class	Locoregional therapy	9
Ray et al. (79) (2023)	United States	Qualitative study	Understanding the barriers and facilitators to hepatocellular carcinoma care.	19	HCC	BLCL class	No active antitumor treatment	9
Li et al. (80) (2021)	China	Qualitative study	Understanding the illness trajectory and self-management experiences of liver cancer patients undergoing TACE.	12	PLC	Not reported	Locoregional therapy	9
Zhang (81) (2015)	China	Qualitative study	The impact of malignant liver tumors on the quality of life of patients.	42	Liver cancer	Not reported	Locoregional therapy; systemic therapy; others	7
Niu et al. (82) (2014)	China	Qualitative study	The experiences of self-perceived burden in liver cancer patients after TACE.	12	Liver cancer	Not reported	Locoregional therapy	8

PROM, patient-reported outcome measure; COST, Comprehensive Score for FT—Functional Assessment of Chronic Illness Therapy; EORTC QLQ-C30, European Organisation for Research and Treatment of Cancer Quality of Life Questionnaire-Core 30; PLC, primary liver cancer; HADS, Hospital Depression and Anxiety Scale; HCC, hepatocellular carcinoma; TNM, TNM stage; BCLC, Barcelona Clinic Liver Cancer Staging; COST-FACIT-V2, Comprehensive Score for FT—Functional Assessment of Chronic Illness Therapy, Version 2; SPBS-CP, Self-Perceived Burden Scale for Cancer Patients; ICC, Intrahepatic cholangiocarcinoma; cHCC-CCA, combined hepatocellular-cholangiocarcinoma; CD-RISC, Connor–Davidson Resilience Scale; mUICC, modified Union for International Cancer Control.

The definition of FT varied considerably across these studies. One study (15) employed numerical thresholds, defining FT as a household’s total out-of-pocket health payments ≥40% of its capacity to pay. The majority of studies reported direct medical costs (29/39, 74.36%). Other dimensions were reported less frequently: indirect costs in 17 studies (43.59%), direct non-medical costs in 13 (33.33%), and financial distress in 16 (41.03%). Only 13 studies (33.33%) integrated all four core FT dimensions. Furthermore, 10 studies (26.64%) reported patients’ quality of life but failed to explore the associated financial factors.

This review systematically examined the instruments used to assess patient-reported FT. The choice of assessment tools varied considerably across studies and could be grouped into four categories. Ten studies (25.64%) used validated FT-specific instruments, all of which applied the COST. The COST was rated on a 2-point (34–36) or 4-point scale (12–14, 32, 37), while one (26) did not report the cut-off values. It ranged from 0 to 44, with lower scores indicating greater FT. Nevertheless, the cut-off values for severity levels differed across studies. Eighteen studies (46.15%) used financial difficulty items derived from broader quality of life instruments, primarily the EORTC QLQ-C30. The scale has a score range of 0–100, with higher scores indicating a greater financial burden. Two studies (15, 38) used objective measures of financial burden derived from patient surveys or interviews to examine the prevalence of FT. Finally, 10 studies employed qualitative assessments of financial hardship, providing in-depth evaluations of patients’ experiences of financial difficulty.

Different studies have identified various factors contributing to FT. These findings, derived from 25 studies (64.10%), were classified into 9 categories: treatment regimens, financial status, insurance coverage, employment status, psychological factors, family status, financial support, demographic factors, and biochemical markers. Specifically, 16 studies (41.03%) noted that treatment for liver cancer patients may affect their experience of FT. Twelve studies (30.78%) reported that the employment status and insurance status of patients with liver cancer were associated with their FT. The detailed findings are presented in Table 2.

**Table 2:** Summary of FT measurement and factors.

Author (year)	Measurement of FT	Results	Factors
Zou et al. (37) (2025)	The COST was used to measure FT. The score ranges from 0 to 44. The severity of FT is divided into four levels:≥24 is no FT, 17–23 is mild FT, 13–16 is moderate FT, and ≤12 is severe FT.	Mean (SD) COST was 20.36 (3.95).	Gender; age; living conditions; monthly income; time of diagnosis; cancer stage; treatments; other diseases
Tsai et al. (57) (2026)	The EORTC QLQ-C30 was used to measure patients’ financial and psychological burden. The score ranges from 0 to 100, with a higher score representing a greater burden.	Mean (SD) EORTC QLQ-C30 financial burden was 2.61(8.96).	Not reported
Li and Zou (32) (2025)	The COST was used to measure FT. The score ranges from 0 to 44. The severity of FT is divided into four levels: ≥26 is no FT, 14–25 is mild FT, 1–13 is moderate FT, and a total score of 0 is severe FT.	Mean (SD) COST was 23.81(8.79); Mean (SD) EORTC QLQ-C30 financial burden was 55.83 (36.45).	Residence; income; occupation; healthcare financial burden
Zou (12) (2025)	The COST was used to measure FT. The score ranges from 0 to 44. The severity of FT is divided into four levels: ≥26 is no FT, 14–25 is mild FT, 1–13 is moderate FT, and a total score of 0 is severe FT.	Mean (SD) COST was18.37 (11.21); FT rate was 75.4%.	Residence; insurance; payment; education; frequency of interventional treatments
Mao et al. (13) (2026)	The COST was used to measure FT. The score ranges from 0 to 44. The severity of FT is divided into four levels: ≥26 is no FT, 14–25 is mild FT, 1–13 is moderate FT, and a total score of 0 is severe FT.	Mean (SD) COST was 23.93 (8.38); FT rate was 86.2%.	Monthly disposable household;income; residence; number of hospitalizations; extrahepatic metastasis; cancer stage
Lee et al. (14) (2025)	The COST was used to measure FT. The score ranges from 0 to 44. The severity of FT is divided into four levels: ≥26 is no FT, 14–25 is mild FT, 1–13 is moderate FT, and a total score of 0 is severe FT.	Mean (SD) COST was 26 (11); FT rate was 45%.	Age; nationality; language barrier; working status; education; treatment
Zhang et al. (36) (2025)	The COST was used to measure FT. The score ranges from 0 to 44, with a lower score representing higher FT. FT was defined as having a COST ≤25.	Mean (SD) COST was 18.62 (6.80).	Education; residence; insurance; working status; income; cancer stage; treatment participation in a clinical trial;
Liu et al. (35) (2025)	The COST was used to measure FT. The score ranges from 0 to 44, with a lower score representing higher FT. FT was defined as having a COST < 19	The median score of COST was 19 (IQR; 10–25).	Working status; income; treatment; social support
Sun et al. (27) (2025)	The EORTC QLQ-C30 was used to measure patients’ financial and psychological burden. The score ranges from 0 to 100, with a higher score representing a greater burden.	Mean (SD) EORTC QLQ-C30 financial burden was 30.98 (30.56).	Not reported
Zhou and Liu (26) (2024)	The COST was used to measure FT. The score ranges from 0 to 44, with a lower score representing higher FT.	Mean (SD) COST was 22.94 (6.33).	Education; income; working status; duration of illness; treatment; age; gender; residence; insurance; financial situation; number of children; other diseases
Wu et al. (34) (2024)	The COST was used to measure FT. The score ranges from 0 to 44, with a lower score representing higher FT. FT was defined as having a COST ≤22.	Mean (SD) COST was 15.52 (6.62); FT rate was 79.30%.	Other disease; education; insurance; occupation; number of hospitalizations; frequency of interventional treatment
Zhu et al. (30) (2024)	The COST was used to measure FT. The score ranges from 0 to 44, with a lower score representing higher FT. FT levels were determined by latent profile analysis.	Mean (SD) COST was 20.50 (0.35); FT rate was 87.02%.	Age; residence; income; payment; psychological resilience; social support; education; working status; living conditions; targeted therapy; interventional treatment
Lei et al. (38) (2020)	FT was defined as a comprehensive assessment of direct medical costs, direct nonmedical costs, indirect costs, psychological distress, and out-of-pocket costs.	FT rate was 92.20%.	Hospital; age; education; occupation; insurance; income; treatment
Li et al. (62) (2019)	The EORTC QLQ-C30 was used to measure patients’ financial and psychological burden. The score ranges from 0 to 100, with a higher score representing a greater burden.	Mean (SD) EORTC QLQ-C30 financial burden was 51.20 (37.22).	Albumin and alkaline phosphatase showed a weak correlation with financial burden.
Li et al. (63) (2019)	The EORTC QLQ-C30 was used to measure patients’ financial and psychological burden. The score ranges from 0 to 100, with a higher score representing a greater burden.	Mean (SD) EORTC QLQ-C30 financial burden was 51.16 (37.26).	C-reactive protein; the C-reactive protein-to-albumin ratio; the Glasgow Prognostic Score; the Inflammatory Index; and the Prognostic Index were all weakly correlated with FT.
Chie et al. (64) (2017)	The EORTC QLQ-C30 was used to measure patients’ financial and psychological burden. The score ranges from 0 to 100, with a higher score representing a greater burden.	Mean (SD) EORTC QLQ-C30 FT was 17 (31) in Europe and 19 (27) in Asia.	Not reported
Tang et al. (65) (2016)	The EORTC QLQ-C30 was used to measure patients’ financial and psychological status. The score ranges from 0 to 100, with a higher score representing a greater burden.	Mean (SD) EORTC QLQ-C30 FT was 38.7 (2.2).	Not reported
Che et al. (15) (2016)	Household’s total out-of-pocket health payments equal or exceed 40% of the household’s capacity to pay.	FT rate was 65.6%.	Marriage; age; gender; education; occupation; insurance
Li et al. (66) (2025)	The EORTC QLQ-C30 was used to measure patients’ quality of life. The score ranges from 0 to 100, with a higher score representing a greater burden.	Mean (SD) EORTC QLQ-C30 was 51.36 (37.02).	Not reported
George et al. (67) (2025)	The EORTC QLQ-C30 was used to measure patients’ financial and psychological burden. The score ranges from 0 to 100, with a higher score representing a greater burden.	Baseline: Mean (SD) EORTC QLQ-C30 financial burden was 63.0 (37.1); at 2 weeks after treatment: Mean (SD) was 66.7 (36.9); at 3 months after treatment: Mean (SD) was 68.9 (34.4).	Not reported
Ong et al. (68) (2025)	The EORTC QLQ-C30 was used to measure patients’ financial and psychological burden. The score ranges from 0 to 100, with a higher score representing a greater burden.	Baseline: Mean (SD) EORTC QLQ-C30 financial burden was 68.8 (33.3); at 2 weeks after TACE: Mean (SD) was 68.5 (31.3).	Not reported
Shomura et al. (69) (2024)	The EORTC QLQ-C30 was used to measure patients’ quality of life. The score ranges from 0 to 100, with a higher score representing a greater burden.	Baseline: Mean (SD) EORTC QLQ-C30 was 10.8; at 1 month after treatment: Mean (SD) was 18.5; at 2 months after treatment: Mean (SD) was 15.7; at 3 months after treatment: Mean (SD) was 12.4.	Not reported
van Roekelet al (70). (2020)	The EORTC QLQ-C30 was used to measure patients’ quality of life. The score ranges from 0 to 100, with a higher score representing a greater burden.	No financial burden	Not reported
Kim et al. (71) (2020)	The EORTC QLQ-C30 was used to measure patients’ quality of life. The score ranges from 0 to 100, with a higher score representing a greater burden.	Baseline: Mean (SD) EORTC QLQ-C30 was 13.33 (25.67); at 12 weeks: Mean (SD) was 7.77 (16.80).	Not reported
Meier et al. (72) (2015)	The EORTC QLQ-C30 was used to measure patients’ quality of life. The score ranges from 0 to 100, with a higher score representing a greater burden.	Mean (SD) EORTC QLQ-C30 was 59.4(40.4)	Symptom burden
Zhang (73) (2012)	The EORTC QLQ-C30 was used to measure patients’ quality of life. The score ranges from 0 to 100, with a higher score representing a greater burden.	Before surgery: Mean (SD) EORTC QLQ-C30 financial burden was 56.95 (30.20); at 7 days after surgery: Mean (SD) was 87.80 (29.22); at 14 days after surgery: Mean (SD) was 70.76 (30.44).	Not reported
Romero et al. (74) (2008)	The EORTC QLQ-C30 was used to measure patients’ quality of life. The score ranges from 0 to 100, with a higher score representing a greater burden.	Baseline: Mean (SD) EORTC QLQ-C30 financial burden was 6.7; at 1 month: Mean (SD) was 6.9; at 3 months: Mean (SD) was 9.8; at 6 months: Mean (SD) was 5.0.	Not reported
Dasgupta et al. (75) (2008)	The EORTC QLQ-C30 was used to measure patients’ quality of life. The score ranges from 0 to 100, with a higher score representing a greater burden.	Baseline: Mean (SD) EORTC QLQ-C30 financial burden was 18.48 (29.24); at 6 months: Mean (SD) was 17.62 (29.13); at 12 months: Mean (SD) was 15.58 (26.81); at 36–48 months: Mean (SD) was 12.50 (23.57)	Age
Brans et al. (25) (2002)	The EORTC QLQ-C30 was used to measure patients’ quality of life. The score ranges from 0 to 100, with a higher score representing a greater burden.	No financial burden	Not reported
Han et al. (76) (2026)	The financial burden experiences of patients with liver cancer receiving targeted therapy in combination with immunotherapy.	8 of 15 participants (53.3%) mentioned bearing heavy financial burdens and expressed a strong desire for economic assistance at the healthcare policy level.	Insurance
Wu et al. (28) (2025)	The experience of FT in patients with primary liver cancer, including direct medical costs, indirect costs, and psychological distress.	(1) Diminished ability to work as a result of treatment for illness; (2) high cost of treatment; (3) lack of health insurance policy support; (4) feeling guilty because of medical expenses; (5) lack of financial information.	Treatment; insurance; psychological distress; working status
Lu et al. (46) (2025)	Factors of patients with liver cancer toward clinical drug trials, including direct medical costs, direct nonmedical costs, and indirect costs.	(1) The high cost of treatment; (2) lower socioeconomic status; (3) lost revenue because of illness; (4) transportation costs and inadequate health insurance coverage.	Treatment; social supports; working status
Sun et al. (77) (2024)	Financial needs of patients receiving immunotherapy with liver cancer, including direct medical costs, direct nonmedical costs, indirect costs, and psychological distress.	(1) High costs of medication; (2) costs of frequent tests and hospitalizations; (3) travel costs; (4) caregiver lost income due to the extent of support required; (4) limitations of insurance coverage.	Treatment; insurance; working status
Wörns et al. (47) (2024)	The financial and psychological burden of patients with liver cancer. Including direct medical costs, indirect costs, and psychological distress.	(1) Treatment decision-making; (2)diminished ability to work and losing their jobs as a result of treatment for illness; (3) emotional impact of treatment; (4) reimbursement rate.	Treatment; insurance; working status; emotional impact
Yu et al. (78) (2023)	Direct medical costs, indirect costs, and psychological needs of patients with liver cancer following interventional treatment.	(1) The reimbursement rate of medical expenses; (2) worry about unanticipated future treatment costs; (3) direct costs from repeated hospitalizations; (4) lost income of family members.	Treatment; insurance; psychological needs; income
Ray et al. (79) (2023)	Financial factors influencing the care of hepatocellular carcinoma, including direct treatment costs, direct nonmedical costs, indirect costs, and associated psychological distress.	Barriers: travel costs; medical and caregiving costs. Facilitators: support from relatives and friends; financial information needs; transportation assistance; health insurance support.	Treatment; understanding of medical and care policies
Li et al. (80) (2021)	The financial and psychological burden of patients with liver cancer receiving TACE.	(1) Hospitalization and other treatment-related costs; (2) psychological distress due to the long-term nature of the disease; (3) psychological distress (guilt) about the financial impact.	Family status; duration of hospitalization; psychological distress
Zhang (81) (2015)	Financial factors affecting patients with liver tumors.	Direct treatment costs, including chemotherapy, radiotherapy, etc.	Treatment; duration of treatment
Niu et al. (82) (2014)	The financial and psychological burden of patients with liver cancer receiving TACE.	(1) Repeated hospitalizations; (2) low reimbursement rate of medical expenses; (3) psychological burden (worry) about negatively impacting the family.	Frequency of hospitalization; insurance; psychological burden

FT, financial toxicity; SD, standard deviation; IQR, interquartile range; COST, Comprehensive Score for FT, functional assessment of chronic illness therapy; EORTC QLQ-C30, European Organisation for Research and Treatment of Cancer Quality of Life Questionnaire-Core 30; TACE, transarterial chemoembolization.

### Rate of FT

FT rates were calculated in 7 of the included studies (17.95%), all of which were cross-sectional studies. The meta-analysis identified substantial heterogeneity across the studies (*I*^2^ = 97.88%). As a result, a random-effects model was used to estimate the pooled prevalence of FT. The pooled analysis revealed that the overall prevalence of FT in liver cancer patients was 74% (95% CI: 61–85%).

To further explore the sources of heterogeneity, subgroup analysis was conducted considering country income level. It was identified that only one included study was from a high income country (HIC), which reported FT prevalence of 45%. As this subgroup included only one study, the estimate was provided as a contextual reference rather than a formal subgroup comparison. The pooled prevalence of the remaining six studies conducted in low middle income countries (LMICs) was 78% (95% CI: 65–88%; *I*^2^ = 98%) (Figure 2).

**Figure 2:**
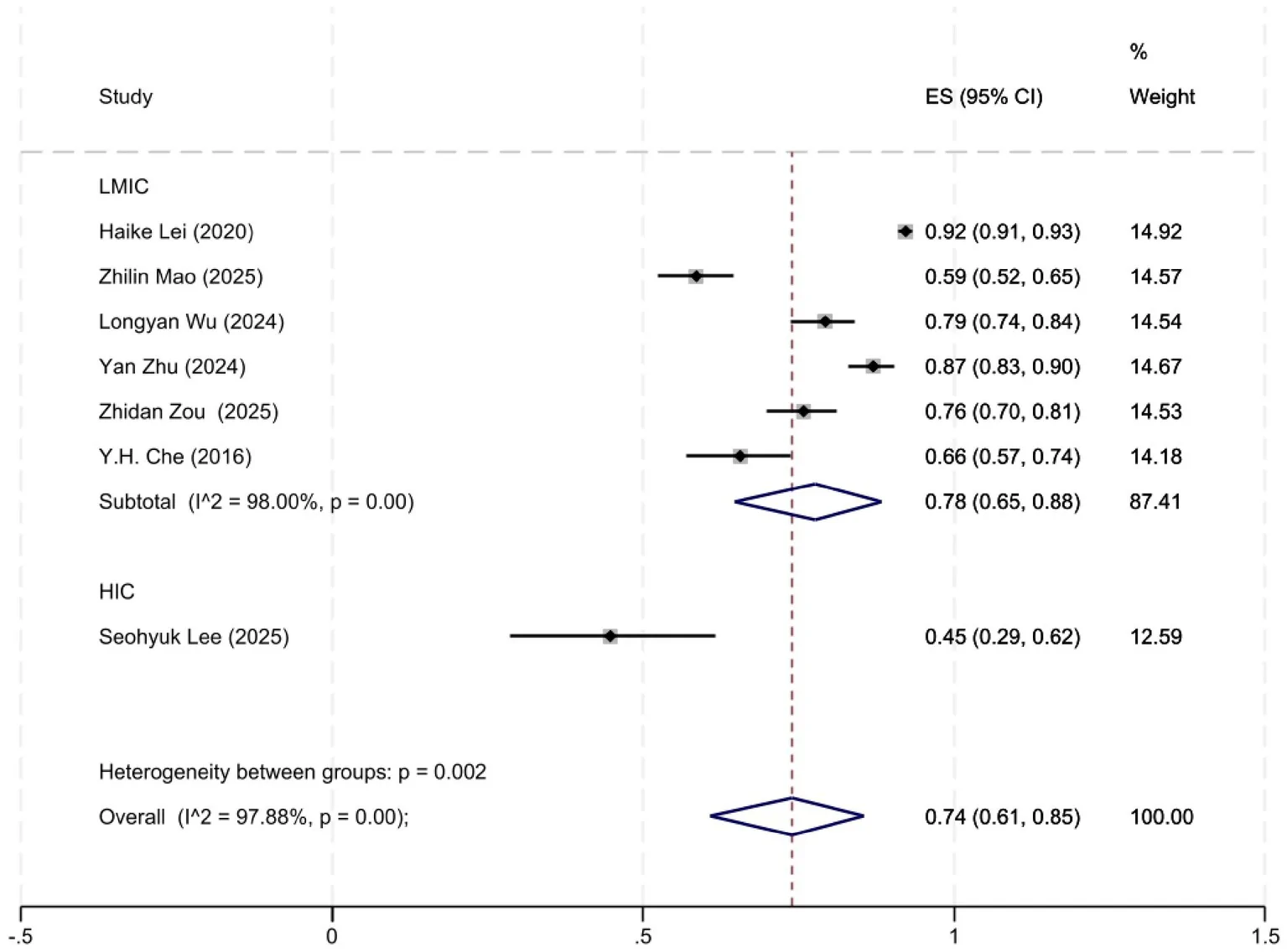
A forest plot on the pooled prevalence of FT among patients with liver cancer.

### Severity of FT

The validated scales were utilized in 27 studies (69.23%) to assess FT in liver cancer patients. Six studies (15.38%) could not be pooled owing to inadequate reporting of outcomes. Ultimately, 21 studies (53.85%) were eligible for meta-analysis, comprising 6 longitudinal studies and 15 cross-sectional studies.

The pooled estimate of FT severity among liver cancer patients in 6 longitudinal studies was 46.32 (95% CI: 26.32–66.32, *I*^2^ = 97%). As all included studies adopted the EORTC QLQ-C30 to assess FT, a subgroup analysis based on country income level was performed. The severity of FT was 30.44 (95% CI: 2.29–58.60, *I*^2^ = 98%) in HICs, and 60.67 (95% CI: 54.41–66.94, *I*^2^ = 5%) in LMICs (Figure 3).

**Figure 3:**
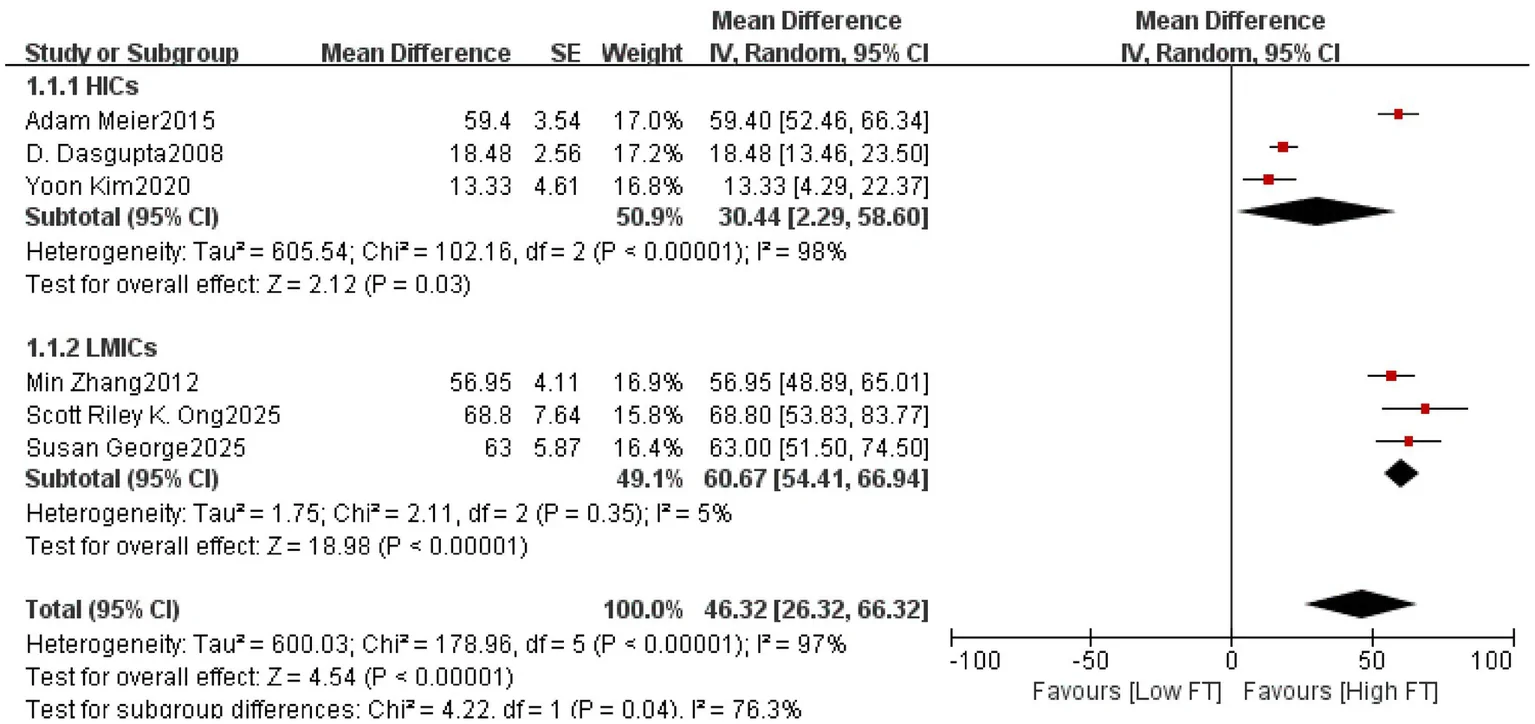
A forest plot on the pooled severity of FT among patients with liver cancer (longitudinal studies).

Of the data from 15 cross-sectional studies, 9 used the COST, and 7 used the EORTC QLQ-C30 to assess FT. One study (32) was incorporated twice into the meta-analysis, given the concurrent use of the two aforementioned scales. Subgroup analysis by country income level was not performed due to the opposite score directions in the two scales. Regarding the measurement of FT, the severity of FT was 20.81 (95% CI: 19.70–21.92, *I*^2^ = 97%) for the COST and 40.18 (95% CI: 27.12–53.24, *I*^2^ = 100%) for the EORTC QLQ-C30 (Figure 4). The results indicate a substantial burden of FT.

**Figure 4:**
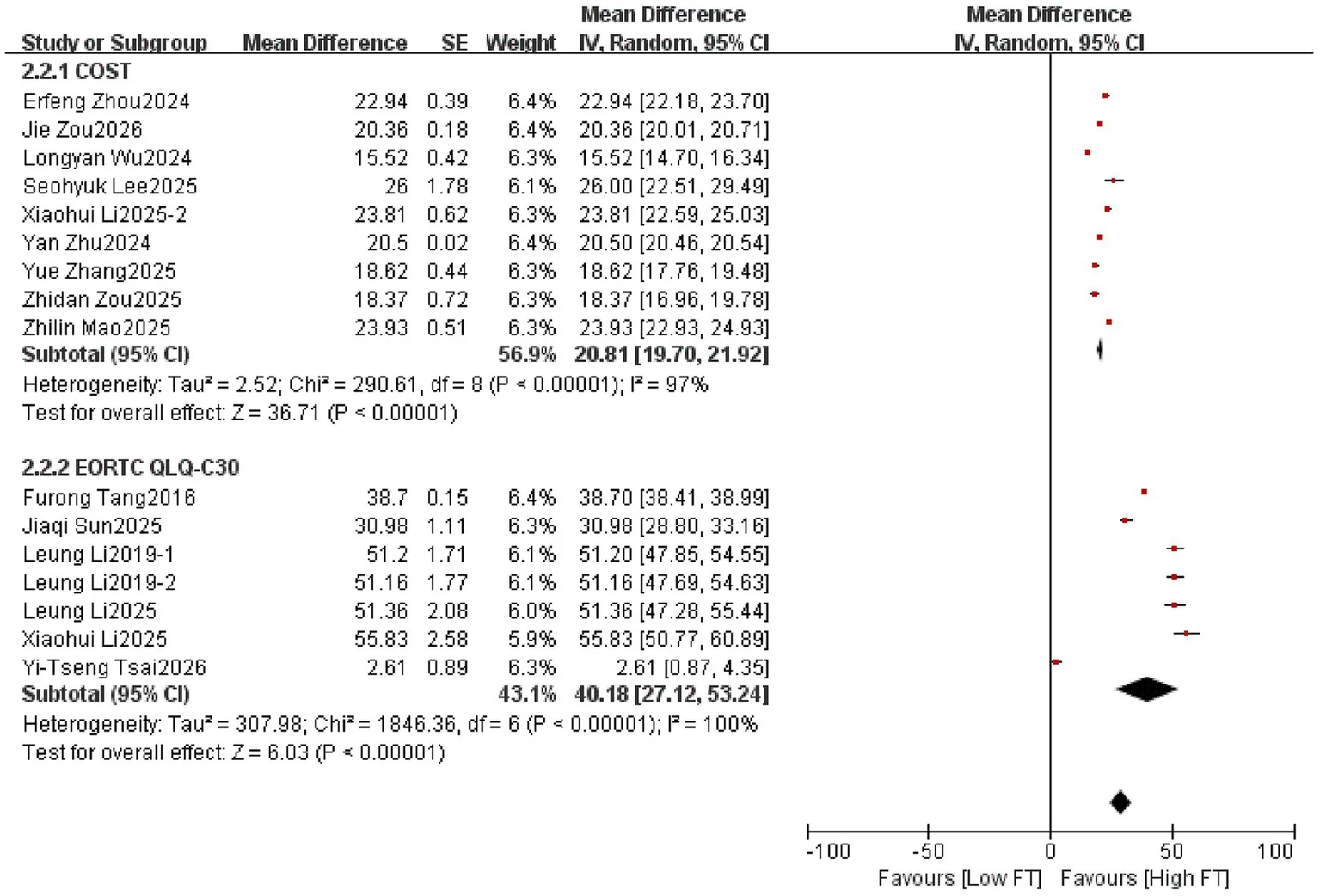
A forest plot on the pooled severity of FT among patients with liver cancer (cross-sectional studies), subgrouped by the measurement tool.

### Publication bias

Analysis of publication bias demonstrated asymmetry in the funnel plot. The Egger’s regression intercept was 18.93 (SE 3.23, 95% CI: 12.00–25.86, *p* < 0.001), suggesting the presence of small-study effects. The trim-and-fill test indicated no study required trimming to correct for plot asymmetry attributed to small-study effects (studies trimmed = 0). Post-adjustment estimates remained equivalent to the unadjusted values. No evidence of significant publication bias was found, and the results remained robust (Figure 5).

**Figure 5:**
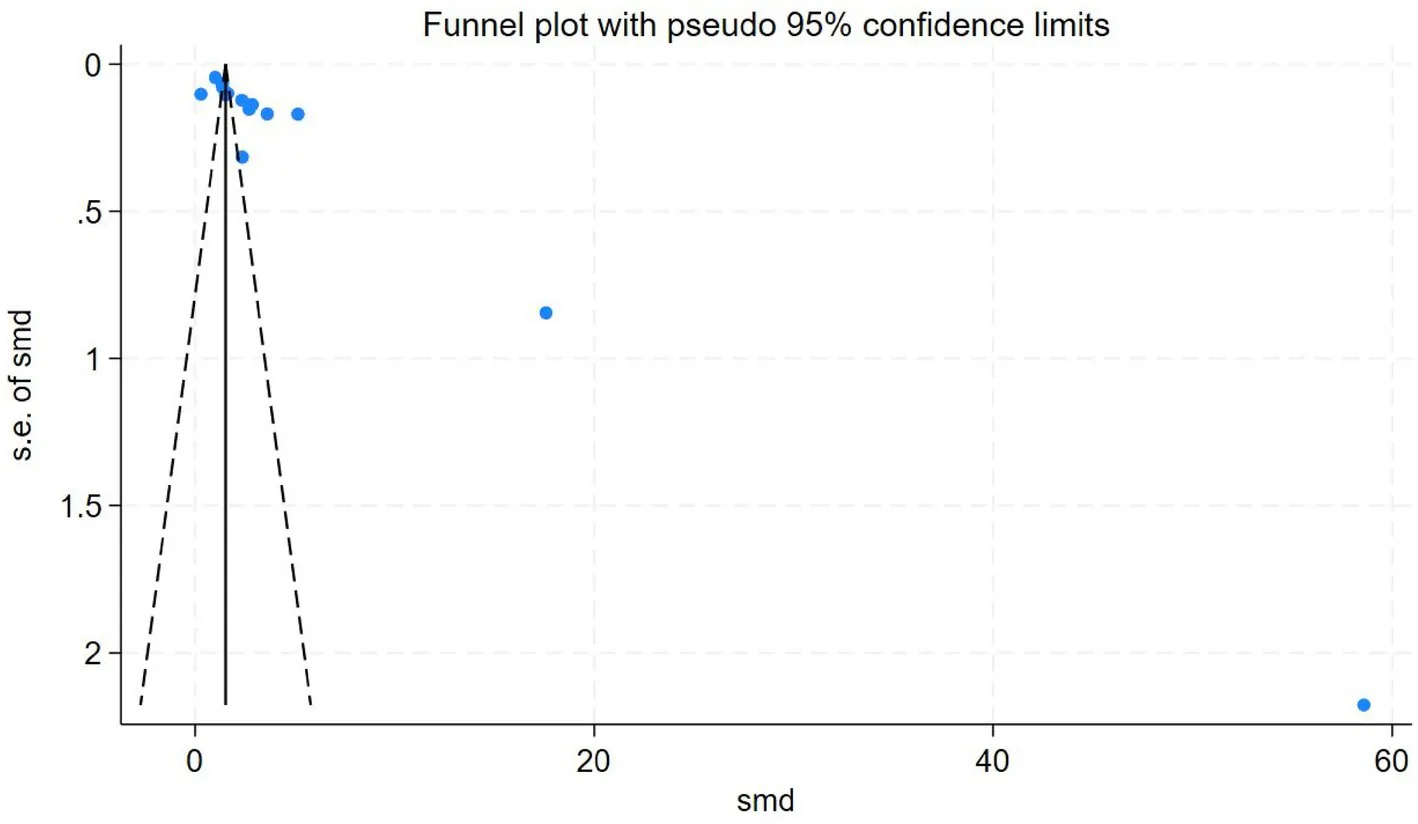
Funnel plot for studies reporting severity of FT among patients with liver cancer.

### Sensitivity analysis

Leave-one-out analysis was carried out to assess the influence of each study on the pooled effect size. This approach sequentially omits one study, and a meta-analysis is performed on the remaining studies. If the CI of the omitted study does not contain the overall effect size estimate (theta), that study is considered to have significant influence on the overall effect size estimate. According to the findings of this study, the pooled effect size remained stable after the omission of any single study.

Leave-one-out sensitivity analysis showed that, in cross-sectional studies, the pooled effect size decreased after excluding Zhu et al. (30) but remained statistically significant (3.16, 95% CI: 1.13–5.19; *p* < 0.05). Heterogeneity did not change substantially, suggesting the pooled effect size remained stable after the omission of any single study (Figures 6–8).

**Figure 6:**
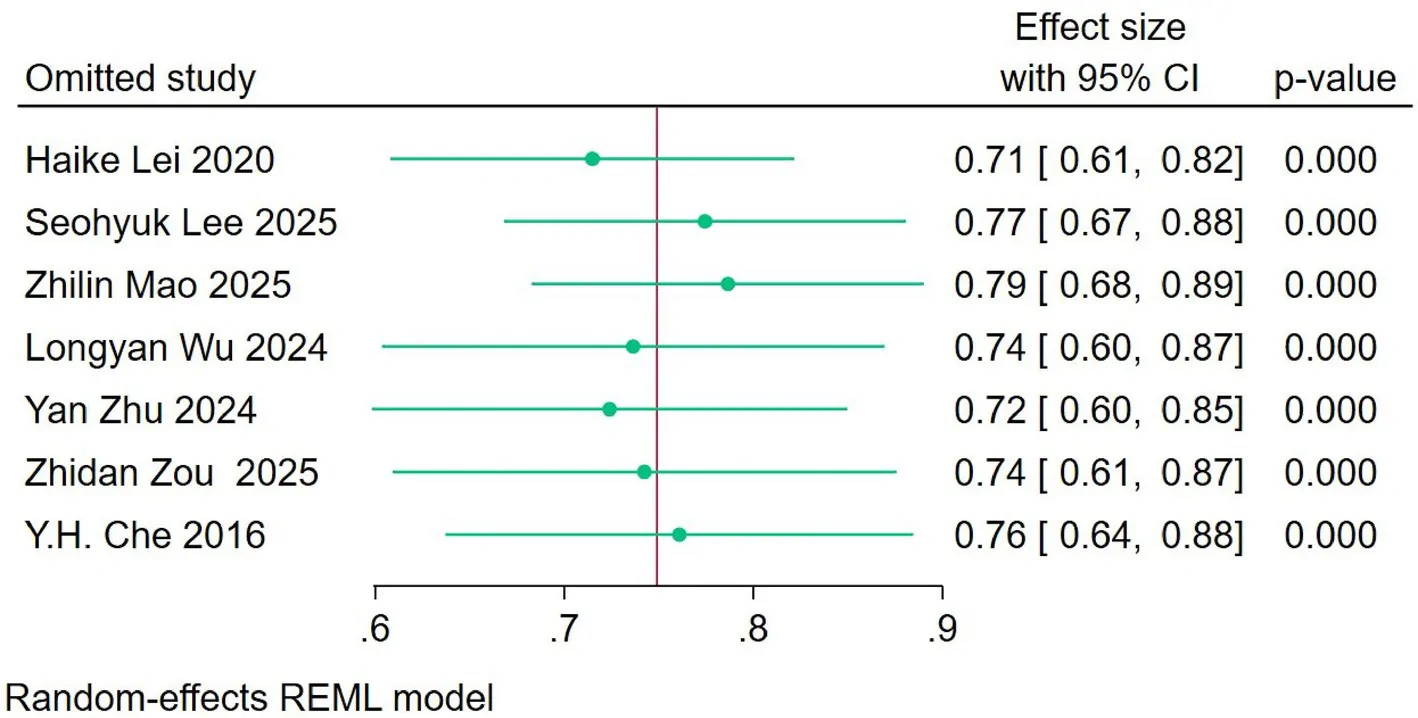
Sensitivity analysis of the pooled rate of FT among patients with liver cancer.

**Figure 7:**
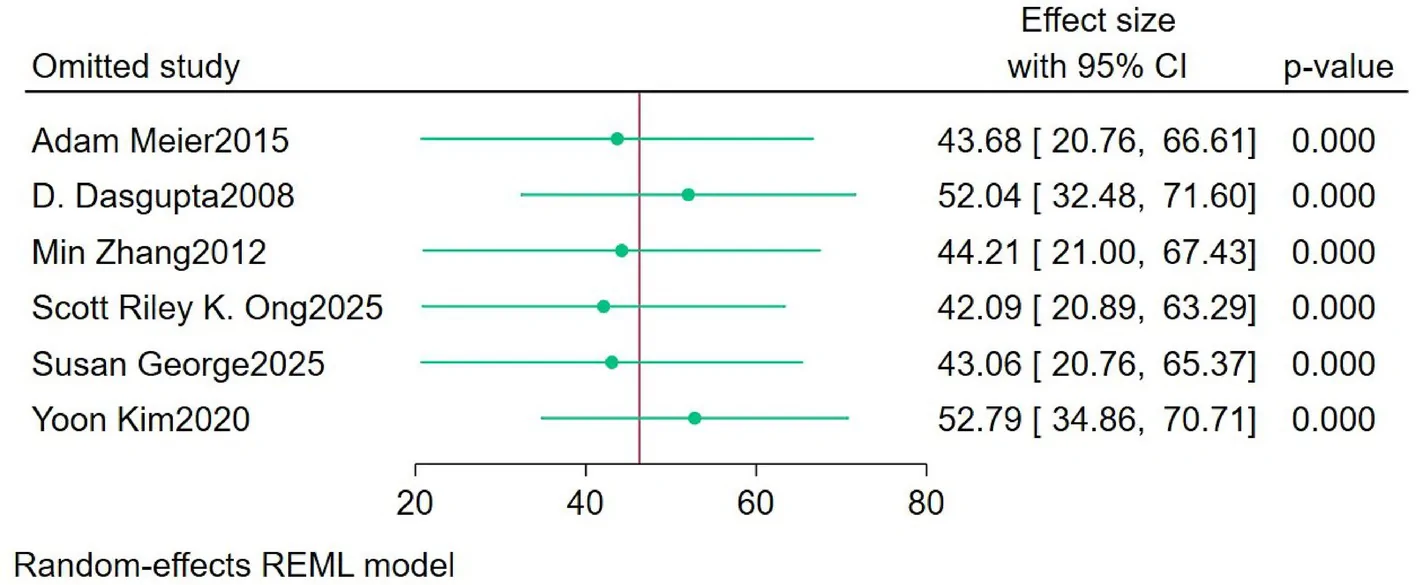
Sensitivity analysis of the severity of FT among patients with liver cancer (longitudinal studies).

**Figure 8:**
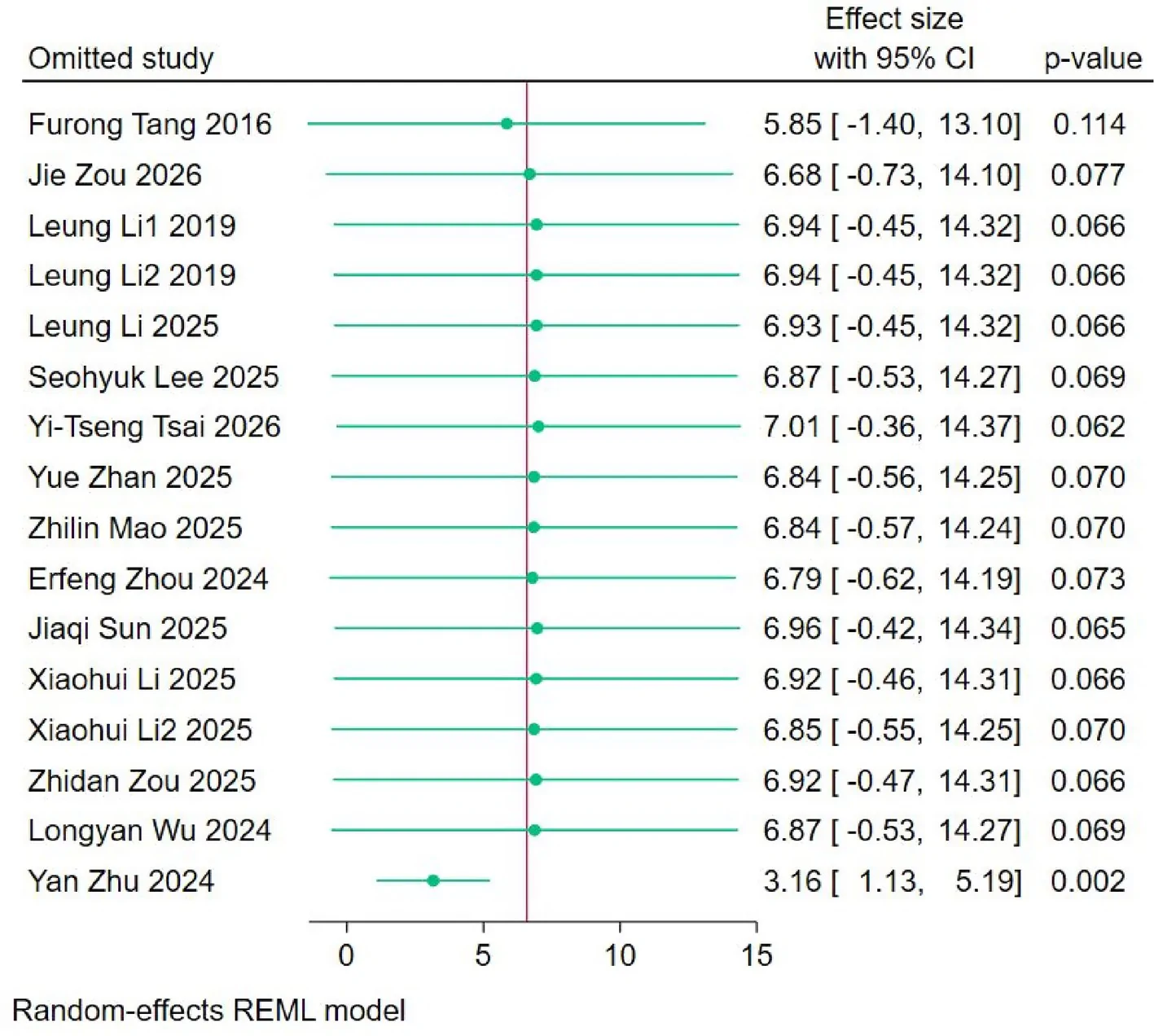
Sensitivity analysis of the severity of FT among patients with liver cancer (cross-sectional studies).

## Discussion

This systematic review and meta-analysis sought to determine the prevalence and severity of FT in patients with liver cancer. The majority of studies (66.67%) were conducted in China, potentially reflecting the large liver cancer population and prevailing policy priorities in China (8). This geographic distribution may also stem from the search strategy of this study, which covered both international and Chinese databases but was restricted to English and Chinese language publications.

Pooled data from 39 eligible studies showed an FT rate of 74.0% in patients with liver cancer. This finding aligns with the FT range of 6–78% reported by Binbin et al. ‘s study (39). In contrast, the results of this study are higher than those reported in a previous systematic review by Jiang et al. (40) in cancer patients (45%).

This study found that the financial strain may be affected by treatment regimens, employment status, and insurance coverage. The cumulative costs of different treatments can be exorbitant, as each treatment regimen incurs separate costs for medications, medical professionals, and hospital stays (29). Moreover, job loss, reduced working hours, impaired work capacity, and other employment disruptions were associated with a higher risk of FT (41). The relationship between negative work changes and FT can be partially attributed to the link between employment and health insurance. In some countries, such as the United States, job loss due to cancer may result in the loss of health insurance (41).

These findings demonstrate that liver cancer treatment still imposes a considerable FT on patients and their families within the current healthcare system. Nevertheless, most existing evidence is derived from patients with diverse treatments (*n* = 23) and lacks comparisons across different insurance status, treatments, and employment status. Therefore, future research should further focus on patients receiving different treatment regimens and insurance coverage. As well as highlights a need for further research to clarify key variables influencing the link between FT and employment, so as to facilitate the development of interventions to reduce FT because of employment change.

The analysis in this study showed a moderately low FT among individuals undergoing liver cancer treatment. This finding is consistent with the range reported in a previous study (42), which indicated FT scores between 10.8 and 26.5 using the COST. In contrast, results of this study are lower than those of a previous systematic review using the COST in cancer patients (20.81) (43).

This result may be influenced by several factors. First, the majority of eligible studies exclusively recruited patients under stable therapeutic management. In contrast, patients who discontinued therapy due to financial pressure were often not included (44, 45). Second, financial pressure may drive patients to adopt coping behaviors (46, 47), including poor treatment adherence, switching to cheaper drugs, or joining clinical trials. Despite alleviating short-term financial burdens, these strategies compromise treatment completion and effectiveness, alongside increasing the risk of adverse clinical outcomes (48). Additionally, insurance coverage may alleviate patients’ financial pressure and further reduce their perceived burden on the scale. Therefore, future studies should pay more attention to patients at different treatment stages and with diverse decision-making styles. High-quality longitudinal studies are also recommended to track dynamic changes of FT during diagnosis and treatment in liver cancer patients. Meanwhile, in clinical practice, medical professionals ought to clearly explain treatment options and associated costs, thereby enabling patients to make fully informed decisions.

The results demonstrated that FT existed in both HICs and LMICs. Subgroup analysis revealed that patients with liver cancer from LMICs experienced a significantly higher level of FT compared with those from HICs. This finding aligns with the previous studies (29, 49). In HICs, this finding may be attributable to the proliferation of novel and costly cancer care interventions, overutilization of health services, longer treatment duration and survivorship care, greater willingness to pay, and variations in insurance coverage (50, 51). Individuals in LMICs often need to finance their medical and non-medical costs by using personal savings, selling assets, skipping bill payments, borrowing, or incurring bank debt (52). Lower baseline income, limited insurance coverage, and greater travel distances for specialized cancer care also contribute (53). This result suggests the urgent need for targeted financial support and health policy reforms to alleviate the FT in patients with liver cancer in LMICs.

This study identified an imbalance in the measurement of FT in liver cancer. Direct medical costs were the most commonly measured dimension (*n* = 29), suggesting that researchers have placed substantial emphasis on direct medical costs. Meanwhile, consistent with the findings from Azzani et al. (54), most included studies (*n* = 22) omitted the evaluation of direct non-medical or indirect costs. This may be due to the difficulty of data acquisition and quantitative calculation. In the absence of such assessment, key stakeholders may fail to pay enough attention to the public health insurance system. As a result, proper policies to support cancer survivors may not be implemented (55). To address this gap, future research is warranted to systematically analyze multi-dimensional financial burden, particularly direct non-medical and indirect costs.

The study found heterogeneity of the assessment tools. Financial burden in liver cancer patients was mainly measured using two scales: the QLQ-C30 and the COST. The QLQ-C30 was widely utilized (*n* = 18) due to its comprehensive assessment of quality of life. The COST, developed exclusively for FT assessment in 2014, originated from the United States and was validated in patients with advanced solid cancers (19). Nevertheless, Thomy’s study pointed out that the COST has prominent limitations in several critical aspects, particularly regarding its internal structure and the interpretation of item domains (56). Similar results were also confirmed in this study. These discrepancies reveal inconsistent criteria for assessing the internal structure of the COST. Meanwhile, the definition of FT varied widely across the included studies. Among ten studies applying the COST, three also used the Hospital Anxiety and Depression Scale (HADS), Self-Perceived Burden Scale for Cancer Patients (SPBS-CP), and Connor–Davidson Resilience Scale (CD-RISC) to assess the psychological burden (30, 36, 57). These tools focus on different aspects, including emotional symptoms, psychological burden, and psychological resilience. These discrepancies are consistent with the findings reported by Witte et al. (58). They pointed out that despite the increasing number of relevant publications, there remained considerable variation and a lack of consensus regarding the interpretation of item domains for subjective financial distress.

These inconsistencies may stem from the absence of a well-defined theoretical framework in the development of the COST as a PROM (58). Without a solid theoretical framework, the COST fails to adequately reflect patients’ actual experiences, indirectly leading to inconsistent assessment standards across FT studies. Currently, several theoretical models have been developed in financial distress. Tucker-Seeley and Thorpe (59) proposed a three-dimensional framework of financial burden, covering material, psychosocial, and behavioral dimensions. Witte et al.’s (58) systematic review summarized six core domains of financial distress: financial spending, financial resources, psychosocial affect, support seeking, coping care, and coping lifestyle. Additionally, other theories and models, such as the Wreckers’ theory of financial distress and the ecological theory, have been widely applied in research among cancer survivors (60, 61). Therefore, future studies are recommended to integrate a theoretical model for FT to revise and optimize the COST by incorporating multiple dimensions of financial distress. In addition, scale validation should establish clinically meaningful cut-off thresholds to standardize the tool’s internal structure and ensure consistent evaluation.

The methodological quality of all cross-sectional and qualitative studies indicated good quality. Cross-sectional studies scored lowest on the item concerning strategies for confounding factors. These findings may compromise the reliability of causal inference, warranting caution when extrapolating the results. Future studies should adequately account for confounding factors at the design stage and prioritize longitudinal designs to verify causal relationships between variables. Qualitative studies scored lowest on the item addressing researcher influence. This finding suggests that the depth and accuracy of understanding patients’ perspectives may have been compromised. Future research should comprehensively document the researcher’s role and reflexive positioning during the study.

The longitudinal studies were rated as moderate. The reason may be that all included longitudinal studies adopted a single-group design and lacked the exposed and unexposed groups required for cohort studies. Additionally, the studies received low scores on the item of follow-up utilization. Most longitudinal studies did not utilize strategies to address incomplete follow-up. This may introduce bias and result in an underestimation of pooled FT severity, as patients with the greatest financial burden and most advanced disease tend to be the earliest to drop out (44). These findings suggest that future studies should adopt prospective long-term follow-up cohort designs to systematically explore the influencing factors of FT in patients with liver cancer.

## Limitations

This study has several limitations. Given the emphasis on patient-centered outcomes of FT and substantial differences in annual incomes across nations, studies that analyzed objective out-of-pocket costs using institutional or insurance databases were excluded. This exclusion may introduce selection bias toward studies of hospital-treated patients. Furthermore, restricting the search to English- and Chinese-language databases may introduce geographic selection bias, yielding a higher proportion of studies conducted in China and potentially limiting the generalizability of findings of this study to global contexts. The predominance of Chinese studies (66.7%) suggests that the pooled estimates of this study largely reflect FT scores under a single healthcare system, thereby constraining their applicability to countries with differing insurance coverage. Moreover, noticeable inconsistencies were observed in the reporting and definition of key variables, such as income, rural/urban status, employment status, cancer stage, comorbidities, and treatment details among the included studies. This may impede standardized comparative analyses and exploration of their specific contributions to FT. Nevertheless, national income status and study quality to mitigate heterogeneity was accounted for when possible.

Future studies should therefore combine objective out-of-pocket cost data from institutional or insurance databases with patient-reported outcomes to enable a more comprehensive assessment of FT. Second, multinational collaborative studies employing standardized data collection protocols are needed to improve cross-national comparability and the generalizability of findings. Third, future systematic reviews should expand the scope of languages and databases searched to enhance the external validity and overall strength of the evidence. Finally, future studies should apply uniform definitions to key variables—such as income, urban/rural status, employment status, cancer stage, comorbidities, and treatment details—to support more robust subgroup analyses and meta-analyses.

## Conclusion

Liver cancer patients present a high prevalence of FT, yet the overall severity remains moderately low. Considerable heterogeneity exists in the assessment tools used across studies, with direct medical costs being the most frequently measured domains. Subgroup analysis revealed that FT burden was significantly higher in LMICs than in HICs. Findings of this study indicate that the lack of standardized measurement tools and inconsistent reporting methods have impaired the comparability of available evidence. The higher FT burden in LMICs underscores an urgent demand for targeted financial support and policy reforms in these regions. Future research should focus on developing a unified and validated FT assessment tool specifically for liver cancer patients, and expand studies in HICs to establish a comprehensive global evidence landscape. In addition, studies on targeted FT intervention are warranted to optimize clinical care and facilitate policy-making in liver cancer care.

## Data Availability

The original contributions presented in the study are included in the article/Supplementary material, further inquiries can be directed to the corresponding author.
