# Supplementary material for: Patient-reported financial toxicity among patients with liver cancer: a systematic review and meta-analysis
Source: Front Public Health. 2026 Oct 15;14:1958294. doi: 10.3389/fpubh.2026.1958294 (PMC13717401; doi:10.3389/fpubh.2026.1958294)
Supplement: Supplementary file 1 [file Table_1.docx]

Supplementary Material

Supplementary file 1 Search strategies used for literature search

Supplementary file 2 Data Sharing Statement

Supplementary file 3 Quality assessment results for the included studies

This supplemental material has been provided by the authors to give readers additional information about their work.

Supplementary file 1 Search strategies used for literature search

| Database | Search strategies | Number of publications |
| --- | --- | --- |
| PubMed  (March 2, 2026) | ((Liver Neoplasms[MeSH Terms]) OR ("Liver Neoplasms, Experimental"[Title/Abstract] OR "Adenoma, Liver Cell"[Title/Abstract] OR "Carcinoma, Hepatocellular"[Title/Abstract] OR "Hepatic Neoplasm*"[Title/Abstract] OR "Neoplasm*, Hepatic"[Title/Abstract] OR "Neoplasm*, Liver"[Title/Abstract] OR "Liver Neoplasm*"[Title/Abstract] OR "Cancer of Liver"[Title/Abstract] OR "Liver Cancer*"[Title/Abstract] OR "Cancer*, Liver"[Title/Abstract] OR "Hepatocellular Cancer*"[Title/Abstract] OR "Cancer*, Hepatocellular"[Title/Abstract] OR "Cancer of the Liver"[Title/Abstract] OR "Cancer*, Hepatocellular"[Title/Abstract] OR "Hepatic Cancer*"[Title/Abstract] OR "Cancer*, Hepatic"[Title/Abstract] OR "Hepatocellular Carcinoma"[Title/Abstract] OR "Experimental Liver Neoplasm*"[Title/Abstract] OR "Liver Neoplasm*, Experimental"[Title/Abstract] OR "Morris Hepatoma*"[Title/Abstract] OR "Novikoff Hepatoma*"[Title/Abstract] OR "Experimental Hepatoma*"[Title/Abstract] OR "Hepatoma*, Experimental"[Title/Abstract])) AND (((Quality of Life[MeSH Major Topic]) OR ("health related quality of life"[Title/Abstract] OR "QoL"[Title/Abstract] OR "HRQoL"[Title/Abstract] OR "cost benefit analysis"[Title/Abstract] OR "cost effectiveness analysis"[Title/Abstract] OR "cost control"[Title/Abstract] OR "health economics"[Title/Abstract] OR "hospital cost"[Title/Abstract] OR "cost minimization analysis"[Title/Abstract] OR "health care cost*"[Title/Abstract] OR "direct cost*"[Title/Abstract] OR "indirect cost*"[Title/Abstract] OR "drug cost*"[Title/Abstract] OR "treatment cost*"[Title/Abstract] OR "health expenditure*"[Title/Abstract] OR "direct expenditure*"[Title/Abstract] OR "out-of-pocket expenditure*"[Title/Abstract] OR "out-of-pocket expense*"[Title/Abstract] OR "out-of-pocket cost*"[Title/Abstract] OR "out-of-pocket payment*"[Title/Abstract] OR "out-of-pocket spending"[Title/Abstract] OR "indirect expenditure*"[Title/Abstract] OR expenditure*[Title/Abstract] OR "cost of treatment"[Title/Abstract] OR "capital expenditure*"[Title/Abstract] OR "direct service cost*"[Title/Abstract] OR "healthcare cost*"[Title/Abstract] OR "medical care cost*"[Title/Abstract] OR "health cost*"[Title/Abstract] OR "financial management*"[Title/Abstract] OR "financial consequence"[Title/Abstract] OR "healthcare expenditure*"[Title/Abstract] OR "health care expenditure*"[Title/Abstract] OR cost of management[Title/Abstract] OR fear of cost[Title/Abstract])) | 2433 |
| CINHAL  (March 2, 2026) | (“Liver Neoplasms” OR “Liver Neoplasms, Experimental” OR “Adenoma, Liver Cell” OR “Carcinoma, Hepatocellular” OR “Hepatic Neoplasm*” OR “Neoplasm*, Hepatic” OR “Neoplasm*, Liver” OR “Liver Neoplasm*” OR “Cancer of Liver” OR “Liver Cancer*” OR “Cancer*, Liver” OR “Hepatocellular Cancer*” OR “Cancer*, Hepatocellular” OR “Cancer of the Liver” OR “Cancer*, Hepatocellular” OR “Hepatic Cancer*” OR “Cancer*, Hepatic” OR “Hepatocellular Carcinoma” OR “Liver Neoplasm*, Experimental”) **AND** (“Financial Stress” OR “Economic Burden*” OR “Financial Burden*” OR “Financial Toxicity*” OR “Financial Challenge*” OR “Financial Pressure*” OR “Financial Strain*” OR “Insufficient Financial Resource*” OR “Financial Resource Strain*” OR “Socioeconomic Adversity*” OR “Financial Hardship*” OR “Economic Hardship*” OR “Quality of life” OR “Health-related quality of life” OR “EORTC QLQ-C30” OR “Cancer Care Outcomes Research and Surveillance Consortium Patient Survey” OR “CanCORS” Or “Social Difficulties Inventory” OR “SDI” OR “the Patient Satisfaction Questionnaire” OR “PSQ-18” OR “The Comprehensive Score for Financial Toxicity” OR “COST” OR “Socioeconomic Wellbeing Scale” OR “SWBS” OR “InCharge Financial Distress” OR “Financial Well-Being Scale” OR “IFDFW” OR “QoL” OR “HRQoL” OR “health related quality of life” OR “cost benefit analysis” OR “cost effectiveness analysis” OR “cost of illness” OR “cost control” OR “health economics” OR “hospital cost” OR “cost minimization analysis” OR “cost of illness” OR “cost of management” OR “health care cost*” OR “direct cost*” OR “indirect cost*” OR “drug cost*” OR “treatment cost*” OR “health expenditure*” OR “direct expenditure*” OR “out of pocket expenditure*” OR “out of pocket expense*" OR “out of pocket cost*” OR “out of pocket payment*” OR “out of pocket spending” OR “indirect expenditure*” OR expenditure* OR “fear of cost*” OR “cost of treatment” OR “capital expenditure*” OR “direct service cost*” OR “healthcare expenditure*” OR “health care expenditure*” OR “healthcare cost*” OR “medical care cost*” OR “health cost*” OR “financial management*” OR “financial consequence”) | 721 |
| Web of Science  (March 2, 2026) | (TS=(“Liver Neoplasms” OR “Liver Neoplasms, Experimental” OR “Adenoma, Liver Cell” OR “Carcinoma, Hepatocellular” OR “Hepatic Neoplasm*” OR “Neoplasm*, Hepatic” OR “Neoplasm*, Liver” OR “Liver Neoplasm*” OR “Cancer of Liver” OR “Liver Cancer*” OR “Cancer*, Liver” OR “Hepatocellular Cancer*” OR “Cancer*, Hepatocellular” OR “Cancer of the Liver” OR “Cancer*, Hepatocellular” OR “Hepatic Cancer*” OR “Cancer*, Hepatic” OR “Hepatocellular Carcinoma” OR “Experimental Liver Neoplasm*” OR “Liver Neoplasm*, Experimental” OR “Hepatoma*, Morris” OR “Morris Hepatoma*” OR “Novikoff Hepatoma*” OR “Experimental Hepatoma*” OR “Hepatoma*, Experimental”)) **AND** (TS=(“Financial Stress” OR “Stress*, Financial” OR “Economic Burden*” OR “Burden, Economic” OR “Financial Burden*” OR “Burden, Financial” OR “Financial Toxicity*” OR “Toxicity, Financial” OR “Financial Challenge*” OR “Challenge*, Financial” OR “Financial Pressure*” OR “Pressure, Financial” OR “Financial Strain*” OR “Strain, Financial” OR “Socioeconomic Adversity*” OR “Adversity, Socioeconomic” OR “Financial Hardship*” OR “Hardship, Financial” OR “Economic Hardship*” OR “Hardship, Economic”OR “Quality of life” OR “health related quality of life” OR “QoL” OR “HRQoL” OR “cost benefit analysis” OR “cost effectiveness analysis” OR “cost of illness” OR “cost control” OR “health economics” OR “hospital cost” OR “cost minimization analysis” OR “cost of management” OR “health care cost*” OR “direct cost*” OR “indirect cost*” OR “drug cost*” OR “treatment cost*” OR “health expenditure*” OR “direct expenditure*” OR “out-of-pocket expenditure*” OR “out-of-pocket expense*” OR “out-of-pocket cost*” OR “out-of-pocket payment*” OR “out-of-pocket spending” OR “indirect expenditure*” OR expenditure* OR “fear of cost*” OR “cost of treatment” OR “capital expenditure*” OR “direct service cost*” OR “healthcare cost*” OR “medical care cost*” OR “health cost*” OR “financial management*” OR “financial consequence” OR “healthcare expenditure*” OR “health care expenditure*” OR “cost of illness”)) | 3127 |
| Embase  (March 2, 2026) | (('liver neoplasms':ti,ab,kw OR 'liver neoplasms, experimental':ti,ab,kw OR 'adenoma, liver cell':ti,ab,kw OR 'carcinoma, hepatocellular':ti,ab,kw OR 'hepatic neoplasm':ti,ab,kw OR 'neoplasm, hepatic':ti,ab,kw OR 'neoplasm, liver':ti,ab,kw OR 'liver neoplasm':ti,ab,kw OR 'cancer of liver':ti,ab,kw OR 'liver cancer':ti,ab,kw OR 'cancer, liver':ti,ab,kw OR 'hepatocellular cancer':ti,ab,kw OR 'cancer of the liver':ti,ab,kw OR 'cancer, hepatocellular':ti,ab,kw OR 'hepatic cancer':ti,ab,kw OR 'cancer, hepatic':ti,ab,kw OR 'hepatocellular carcinoma':ti,ab,kw OR 'experimental liver neoplasm':ti,ab,kw OR 'liver neoplasm, experimental':ti,ab,kw OR 'hepatoma, morris':ti,ab,kw OR 'morris hepatoma':ti,ab,kw OR 'hepatoma, novikoff':ti,ab,kw OR 'novikoff hepatoma':ti,ab,kw OR 'experimental hepatoma':ti,ab,kw OR 'hepatoma, experimental':ti,ab,kw) **AND** ('financial stress':ti,ab,kw OR 'stress, financial':ti,ab,kw OR 'economic burden':ti,ab,kw OR 'burden, economic':ti,ab,kw OR 'financial burden':ti,ab,kw OR 'burden, financial':ti,ab,kw OR 'financial toxicity':ti,ab,kw OR 'toxicity, financial':ti,ab,kw OR 'financial challenge':ti,ab,kw OR 'challenge, financial':ti,ab,kw OR 'financial pressure':ti,ab,kw OR 'pressure, financial':ti,ab,kw OR 'financial strain':ti,ab,kw OR 'strain, financial':ti,ab,kw OR 'socioeconomic adversity':ti,ab,kw OR 'adversity, socioeconomic':ti,ab,kw OR 'financial hardship':ti,ab,kw OR 'hardship, financial':ti,ab,kw OR 'economic hardship':ti,ab,kw OR 'hardship, economic':ti,ab,kw OR 'quality of life':ti,ab,kw OR 'health related quality of life':ti,ab,kw OR 'qol':ti,ab,kw OR 'hrqol':ti,ab,kw OR 'cost benefit analysis':ti,ab,kw OR 'cost effectiveness analysis':ti,ab,kw OR 'cost of illness':ti,ab,kw OR 'cost control':ti,ab,kw OR 'health economics':ti,ab,kw OR 'hospital cost':ti,ab,kw OR 'cost minimization analysis':ti,ab,kw OR 'cost of management':ti,ab,kw OR 'health care cost':ti,ab,kw OR 'direct cost':ti,ab,kw OR 'indirect cost':ti,ab,kw OR 'drug cost':ti,ab,kw OR 'treatment cost':ti,ab,kw OR 'health expenditure':ti,ab,kw OR 'direct expenditure':ti,ab,kw OR 'out of pocket expenditure':ti,ab,kw OR 'out of pocket expense':ti,ab,kw OR 'out of pocket cost':ti,ab,kw OR 'out of pocket payment':ti,ab,kw OR 'out of pocket spending':ti,ab,kw OR 'indirect expenditure':ti,ab,kw OR expenditure:ti,ab,kw OR 'fear of cost':ti,ab,kw OR 'cost of treatment':ti,ab,kw OR 'capital expenditure':ti,ab,kw OR 'direct service cost':ti,ab,kw OR 'healthcare expenditure':ti,ab,kw OR 'healthcare cost':ti,ab,kw OR 'medical care cost':ti,ab,kw OR 'health cost':ti,ab,kw OR 'financial management':ti,ab,kw OR 'financial consequence':ti,ab,kw)) NOT review | 3701 |
| Cochrane  (March 2, 2026) | (“Liver Neoplasms” OR “Liver Neoplasms, Experimental” OR “Adenoma, Liver Cell” OR “Carcinoma, Hepatocellular” OR “Hepatic Neoplasm” OR “Neoplasm, Hepatic” OR “Neoplasm, Liver” OR “Liver Neoplasm” OR “Cancer of Liver” OR “Liver Cancer” OR “Cancer, Liver” OR “Hepatocellular Cancer” OR “Cancer, Hepatocellular” OR “Cancer of the Liver” OR “Cancer, Hepatocellular” OR “Hepatic Cancer” OR “Cancer, Hepatic” OR “Hepatocellular Carcinoma”) **AND** (“Financial Stress” OR “Stress, Financial” OR “Economic Burden” OR “Burden, Economic” OR “Financial Burden” OR “Burden, Financial” OR “Financial Toxicity” OR “Toxicity, Financial” OR “Financial Challenge” OR “Challenge, Financial” OR “Financial Pressure” OR “Pressure, Financial” OR “Financial Strain” OR “Strain, Financial” OR “Socioeconomic Adversity” OR “Adversity, Socioeconomic” OR “Financial Hardship” OR “Hardship, Financial” OR “Economic Hardship” OR “Hardship, Economic” OR “Quality of life” OR “health related quality of life” OR “QoL” OR “HRQoL” OR “cost benefit analysis” OR “cost effectiveness analysis” OR “cost of illness” OR “cost control” OR “health economics” OR “hospital cost” OR “cost minimization analysis” OR “cost of management” OR “health care cost” OR “direct cost” OR “indirect cost” OR “drug cost” OR “treatment cost” OR “health expenditure” OR “direct expenditure” OR “out of pocket expenditure” OR “out of pocket expense” OR “out of pocket cost” OR “out of pocket payment” OR “out of pocket spending” OR “indirect expenditure” OR expenditure OR “fear of cost” OR “cost of treatment” OR “capital expenditure” OR “direct service cost” OR “healthcare expenditure” OR “healthcare cost” OR “medical care cost” OR “health cost” OR “financial management” OR “financial consequence”) | 1164 |
| CNKI  (March 2, 2026) | （主题=肝肿瘤 + 肝部肿瘤 + 肝脏肿瘤 + 肝细胞癌症 + 肝细胞癌 + 肝癌 + “癌，肝细胞” + 肝细胞瘤 + “腺瘤，肝细胞” + 肝细胞腺瘤 + 肝细胞性腺瘤） **AND** （主题=经济毒性 + 财务毒性 + 金融毒性 + 经济负担 + 财务负担 + 经济困难 + 财务困难 + 财务困境 + 经济压力 + 财务压力 + 健康相关生活质量 + 财务管理 + 自付费用 + 自费支出 + 治疗费用 + 治疗成本 + 医疗费用 + 医疗保健费用 + 医疗保健成本 + 卫生保健费用 + 卫生保健支出 + 卫生保健成本） | 321 |
| Wanfang Data  (March 2, 2026) | 题名或关键词:(肝肿瘤 or 肝部肿瘤 or 肝脏肿瘤 or 肝细胞癌症 or 肝细胞癌 or 肝癌 or “癌，肝细胞” or 肝细胞瘤 or 实验性肝细胞瘤 or “腺瘤，肝细胞”or 肝细胞性腺瘤 or 良性肝细胞瘤 or 肝细胞腺瘤) **and** 题名或关键词:(经济毒性 or 财务毒性 or 金融毒性 or 经济负担 or 财务负担 or 经济困难 or 财务困难 or 财务困境 or经济压力 or 财务压力 or 健康相关生活质量 or 财务管理 or 自付费用 or 自费支出 or 治疗费用 or 治疗成本 or 医疗费用 or 医疗保健费用 or 医疗保健成本 or 卫生保健费用 or 卫生保健支出 or 卫生保健成本) | 392 |
| Sinomed  (March 2, 2026) | ( "肝脏肿瘤"[常用字段:智能] OR "肝癌"[常用字段:智能] OR "肝细胞癌症"[常用字段:智能] OR "肝细胞癌瘤"[常用字段:智能] OR "肝部恶性肿瘤"[常用字段:智能] OR "肝脏恶性肿瘤"[常用字段:智能] OR "肝恶性肿瘤"[常用字段:智能] OR "肝部癌症"[常用字段:智能] OR "肝脏癌症"[常用字段:智能] OR "肝肿瘤"[常用字段:智能] OR "肝部肿瘤"[常用字段:智能] OR "成人肝细胞癌"[常用字段:智能] OR "成人肝癌"[常用字段:智能] OR "肝细胞瘤"[常用字段:智能] OR "癌, 肝细胞"[常用字段:智能]) AND( "经济毒性"[常用字段:智能] OR "财务毒性"[常用字段:智能] OR "金融毒性"[常用字段:智能] OR "经济负担"[常用字段:智能] OR "财务负担"[常用字段:智能] OR "经济困难"[常用字段:智能] OR "财务困难"[常用字段:智能] OR "财务困境"[常用字段:智能] OR "经济压力"[常用字段:智能] OR "财务压力"[常用字段:智能] OR "财务管理"[常用字段:智能] OR "自付费用"[常用字段:智能] OR "自费支出"[常用字段:智能] OR "治疗费用"[常用字段:智能] OR "治疗成本"[常用字段:智能] OR "医疗费用"[常用字段:智能] OR "医疗保健费用"[常用字段:智能] OR "医疗保健成本"[常用字段:智能] OR "卫生保健费用"[常用字段:智能] OR "卫生保健支出"[常用字段:智能] OR "卫生保健成本"[常用字段:智能]) | 559 |
| VIP  (March 2, 2026) | （任意字段=肝肿瘤 OR 肝部肿瘤 OR 肝脏肿瘤 OR 肝细胞癌症 OR 肝细胞癌 OR 肝癌 OR “肝肿瘤, 实验性” OR “癌，肝细胞” OR 肝细胞瘤 OR 成人肝癌 OR Novikoff肝细胞瘤 OR 实验性肝细胞瘤 OR Morris肝细胞瘤 OR 实验性肝肿瘤 OR “腺瘤，肝细胞” OR 肝细胞腺瘤 OR 肝细胞性腺瘤 OR 良性肝细胞瘤） AND （任意字段=经济毒性 OR 财务毒性 OR 金融毒性 OR 经济负担 OR 财务负担 OR 经济困难 OR 财务困难 OR 财务困境 OR 经济压力 OR 财务压力 OR 健康相关生活质量 OR 财务管理 OR 自付费用 OR 自费支出 OR 治疗费用 OR 治疗成本 OR 医疗费用 OR 医疗保健费用 OR 医疗保健成本 OR 卫生保健费用 OR 卫生保健支出 OR 卫生保健成本） | 994 |

Supplementary file 2 Data Sharing Statement

**Data Sharing Statement**

**Data**

**Data available:** No

**Additional Information**

**Explanation for why data not available:** All extracted and calculated data are available by emailing to the corresponding author on reasonable request

Supplementary file 3 Quality assessment results for the included studies

| Quality assessment results for cross-sectional studies | | | | | | | | | |
| --- | --- | --- | --- | --- | --- | --- | --- | --- | --- |
| Author(Year) | Item 1 | Item 2 | Item 3 | Item 4 | Item 5 | Item 6 | Item 7 | Item 8 | Total |
| Jie Zou (2026) | 1 | 1 | 1 | 1 | 1 | 1 | 1 | 1 | 8 |
| YiTseng Tsai (2026) | 1 | 1 | 1 | 1 | 1 | 1 | 1 | 1 | 8 |
| Xiaohui Li(2025) | 1 | 1 | 1 | 1 | 1 | 1 | 1 | 1 | 8 |
| Zhidan Zou(2025) | 1 | 1 | 1 | 1 | 1 | 1 | 1 | 1 | 8 |
| Zhilin Mao(2025) | 1 | 1 | 1 | 1 | 1 | 1 | 1 | 1 | 8 |
| Seohyuk Lee(2025) | 1 | 1 | 1 | 1 | 1 | 1 | 1 | 1 | 8 |
| Yue Zhang(2025) | 1 | 1 | 1 | 1 | 1 | 0 | 1 | 1 | 7 |
| Shuyue Liu(2025) | 1 | 1 | 1 | 1 | 1 | 1 | 1 | 1 | 8 |
| Leung Li(2025) | 1 | 1 | 1 | 1 | 1 | 1 | 1 | 1 | 8 |
| Jiaqi Sun(2025) | 1 | 1 | 1 | 1 | 1 | 1 | 1 | 1 | 8 |
| Erfeng Zhou(2024) | 1 | 1 | 1 | 1 | 1 | 1 | 1 | 1 | 8 |
| Longyan Wu(2024) | 1 | 1 | 1 | 1 | 1 | 1 | 1 | 1 | 8 |
| Yan Zhu(2024) | 1 | 1 | 1 | 1 | 1 | 0 | 1 | 1 | 7 |
| Haike Lei(2020) | 1 | 1 | 0 | 1 | 1 | 1 | 0 | 1 | 6 |
| Leung Li(2019) | 1 | 1 | 1 | 1 | 1 | 1 | 1 | 1 | 8 |
| Leung Li(2019) | 1 | 1 | 1 | 1 | 1 | 1 | 1 | 1 | 8 |
| WeiChu CHIE(2017) | 1 | 1 | 1 | 1 | 1 | 1 | 1 | 1 | 8 |
| Furong Tang(2016) | 1 | 1 | 1 | 1 | 0 | 0 | 1 | 1 | 6 |
| YH Che(2016) | 1 | 1 | 0 | 1 | 1 | 1 | 0 | 1 | 6 |

Notes: Item 1: Were the criteria for inclusion in the sample clearly defined; Item 2: Were the study subjects and the setting described in detail; Item 3: Was the exposure measured in a valid and reliable way; Item 4: Were objective, standard criteria used for measurement of the condition; Item 5: Were confounding factors identified; Item 6: Were strategies to deal with confounding factors stated; Item 7: Were the outcomes measured in a valid and reliable way; Item 8: Was appropriate statistical analysis used

| Quality assessment results for longitudinal studies | | | | | | | | | | | | |
| --- | --- | --- | --- | --- | --- | --- | --- | --- | --- | --- | --- | --- |
| Author (Year) | Item 1 | Item 2 | Item 3 | Item 4 | Item 5 | Item 6 | Item 7 | Item 8 | Item 9 | Item 10 | Item 11 | Total |
| Susan George(2025) | 0 | 0 | 1 | 1 | 1 | 0 | 1 | 1 | 1 | 0 | 1 | 7 |
| Scott Riley K. Ong(2025) | 0 | 0 | 1 | 1 | 0 | 0 | 1 | 1 | 1 | 0 | 1 | 6 |
| Masako Shomura(2024) | 0 | 0 | 1 | 1 | 0 | 1 | 1 | 1 | 1 | 1 | 1 | 8 |
| Caren van Roekel(2020) | 0 | 0 | 1 | 1 | 1 | 1 | 1 | 1 | 1 | 1 | 1 | 9 |
| Yoon Kim(2020) | 0 | 0 | 1 | 1 | 0 | 1 | 1 | 1 | 1 | 0 | 1 | 7 |
| Adam Meier(2015) | 0 | 0 | 1 | 1 | 1 | 1 | 1 | 1 | 1 | 0 | 1 | 8 |
| Min Zhang(2012) | 0 | 0 | 1 | 1 | 1 | 0 | 1 | 1 | 0 | 0 | 1 | 6 |
| Méndez Romero A(2008) | 0 | 0 | 1 | 1 | 0 | 0 | 1 | 1 | 1 | 0 | 1 | 6 |
| D. Dasgupta(2008) | 0 | 0 | 1 | 1 | 1 | 0 | 1 | 1 | 1 | 0 | 1 | 7 |
| B. Brans(2002) | 0 | 0 | 1 | 1 | 1 | 0 | 1 | 1 | 1 | 0 | 1 | 7 |

Notes: Item 1 Is there congruity between the stated philosophical perspective and the research methodology; Item 2 Is there congruity between the research methodology and the research question or objectives; Item 3 Is there congruity between the research methodology and the methods used to collect data; Item 4 Is there congruity between the research methodology and the representation and analysis of data; Item 5 Is there congruity between the research methodology and the interpretation of results; Item 6 Is there a statement locating the researcher culturally or theoretically; Item 7 Is the influence of the researcher on the research, and vice versa, addressed; Item 8 Are participants, and their voices, adequately represented; Item 9 Is the research ethical according to current criteria or, for recent studies, and is there evidence of ethical approval by an appropriate body; Item 10 Do the conclusions drawn in the research report flow from the analysis, or interpretation, of the data?

| Quality assessment results for qualitative studies | | | | | | | | | | | |
| --- | --- | --- | --- | --- | --- | --- | --- | --- | --- | --- | --- |
| Author(Year) | Item 1 | Item 2 | Item 3 | Item 4 | Item 5 | Item 6 | Item 7 | Item 8 | Item 9 | Item 10 | Total |
| Bing Han(2026) | 1 | 1 | 1 | 1 | 1 | 1 | 1 | 1 | 1 | 1 | 10 |
| Yanfei Wu(2025) | 1 | 1 | 1 | 1 | 1 | 1 | 0 | 1 | 1 | 1 | 9 |
| Wen Lu(2025) | 1 | 1 | 1 | 1 | 1 | 1 | 1 | 1 | 1 | 1 | 10 |
| Qing Sun(2024) | 1 | 1 | 1 | 1 | 1 | 1 | 0 | 1 | 1 | 1 | 9 |
| Marcus Alexander Wörns(2024) | 1 | 1 | 1 | 1 | 1 | 1 | 1 | 1 | 1 | 1 | 10 |
| Renyue Yu (2023) | 1 | 1 | 1 | 1 | 1 | 1 | 0 | 1 | 1 | 1 | 9 |
| Emily M. Ray(2023) | 1 | 1 | 1 | 1 | 1 | 1 | 0 | 1 | 1 | 1 | 9 |
| Xuening Li(2021) | 1 | 1 | 1 | 1 | 1 | 1 | 0 | 1 | 1 | 1 | 9 |
| Xiuhong Zhang(2015) | 1 | 1 | 1 | 1 | 1 | 1 | 0 | 0 | 0 | 1 | 7 |
| Xiaoxia Niu(2014) | 1 | 1 | 1 | 1 | 1 | 1 | 0 | 1 | 0 | 1 | 8 |

Notes: Item 1 Were the two groups similar and recruited from the same population; Item 2 Were the exposures measured similarly to assign people to both exposed and unexposed groups; Item 3 Was the exposure measured in a valid and reliable way; Item 4 Were confounding factors identified; Item 5 Were strategies to deal with confounding factors stated; Item 6 Were the groups/participants free of the outcome at the start of the study (or at the moment of exposure); Item 7 Were the outcomes measured in a valid and reliable way; Item 8 Was the follow up time reported and sufficient to be long enough for outcomes to occur; Item 9 Was follow up complete, and if not, were the reasons to loss to follow up described and explored; Item 10 Were strategies to address incomplete follow up utilized; Item11 Was appropriate statistical analysis used?
